# Characterizing rare fluctuations in soft particulate flows

**DOI:** 10.1038/s41467-017-00022-8

**Published:** 2017-04-10

**Authors:** S.H.E. Rahbari, A.A. Saberi, Hyunggyu Park, J. Vollmer

**Affiliations:** 1 School of Physics, Korea Institute for Advanced Study, Seoul, 130-722 South Korea; 2 Department of Physics, College of Science, University of Tehran, 14395-547, Tehran, Iran; 3 School of Physics and Accelerators, Institute for research in Fundamental Science (IPM), 19395-5531, Tehran, Iran; 4 Institut für Theoretische Physik, Universitat zu Köln, Zülpicher Strasse 77, 50937 Köln, Germany; 5 Max Planck Institute for Dynamics and Self-Organization (MPI DS), Göttingen, 37077 Germany; 6 Faculty of Physics, Georg August University Göttingen, Göttingen, 37077 Germany; 7 Faculty of Mathematics and Computer Sciences, Georg August University Göttingen, Göttingen, 37073 Germany; 8 Politecnico di Torino, Department of Mathematical Sciences, Corso Duca degli Abruzzi, 24, 10129 Torino, Italy

## Abstract

Soft particulate media include a wide range of systems involving athermal dissipative particles both in non-living and biological materials. Characterization of flows of particulate media is of great practical and theoretical importance. A fascinating feature of these systems is the existence of a critical rigidity transition in the dense regime dominated by highly intermittent fluctuations that severely affects the flow properties. Here, we unveil the underlying mechanisms of rare fluctuations in soft particulate flows. We find that rare fluctuations have different origins above and below the critical jamming density and become suppressed near the jamming transition. We then conjecture a time-independent local fluctuation relation, which we verify numerically, and that gives rise to an effective temperature. We discuss similarities and differences between our proposed effective temperature with the conventional kinetic temperature in the system by means of a universal scaling collapse.

## Abstract (web summary)

Soft particulate flows such as granular media are prone to fluctuations like jamming and avalanches. Here Rahbari *et al.* consider the statistics of rare fluctuations to identify an effective temperature which, unlike previous ones, is valid for packing fractions both near and far from the jamming point.

## Introduction

Large fluctuations are a distinguishing feature of soft particulate flows, like flows of granular media^1, 2^, bubbles and foams^3^, and in living matter such as biological tissues^4, 5^. Very dense systems are in a jammed state. They only move in response to a strong external force. Less packed systems are in a fluid state. They flow in response to any finite force. The flows are highly intermittent and involve rare, very large fluctuations^6^ that can trigger transitions between the jammed and the fluid state^7^. Landslides^8^ and avalanches^9^ are transitions from a jammed to a fluid state. Clogging of hoppers^10^ and breakdown of silos^11^ involve the transition from a fluid to a jammed state. Predicting the frequency of appearance of such fluctuations is a question of great practical and theoretical interest.

Fluctuation relations (FRs) compare the probability of the forward progression of a dynamics and its reverse; akin of watching a movie played in forward and reverse direction. They provide an exact symmetry property of the probability distribution function (PDF) characterizing the likelihood to encounter a given course of states in an observation of the dynamics. Close to equilibrium this symmetry entails linear response. Far from equilibrium FRs have been adopted in micro-biological systems to determine the free energy of a folding RNA^12^ and thermodynamic properties of other biomolecules^13, 14^. In contrast to the dynamics of the microscopic biological systems the dynamics of most macroscopic systems do not move against an exerted force^15^. The strongly fluctuating and intermittent flows of soft particulate matter are a noticeable exception to this rule.

Here, we analyze the statistics of those very large fluctuations where the flow is moving against a driving force. We discuss rare fluctuations in flows of soft particulate matter, where the injected power, *p* = d*w*/d*t ∝ σ*_*xy*_ ⋅ *δv* takes negative values in a finite domain that is subjected to a velocity gradient *δv* and that resists flow by a shear stress *σ*_*xy*_ (the shear stress is the force resisting the flow, see Supplementary Notes 1, 2 and 3). In a steady state the injected energy balances the energy dissipated by the viscosity of the fluid. Hence, on average *p* takes a positive value, and in the thermodynamic limit it does not fluctuate. When there is a finite number of particles in the considered domain there is a small chance to encounter rare fluctuations where *p* takes a negative value. This can either be due to the reversal of the shear stress *σ*_*xy*_ or to the velocity gradient *δv*. While one might naively expect that fluctuations in *σ*_*xy*_ and *δv* would equally contribute to such violations, our numerical simulations show an unexpected interplay of these two mechanisms of rare fluctuations. Moreover, we establish a variation of FR for the statistics of the injected power driving the flow and use it to define an effective temperature for far-from-equilibrium soft particulate flows. Our approach can be easily generalized to study negative power fluctuations and effective temperatures both in simulations and experiments in a wide range of problems such as in the sheared foams, vibrated granular media, particles down an inclined plane, emulsions and other soft particulate media.

## Results

### PDF of injected power *p*

**Fig. 1:**
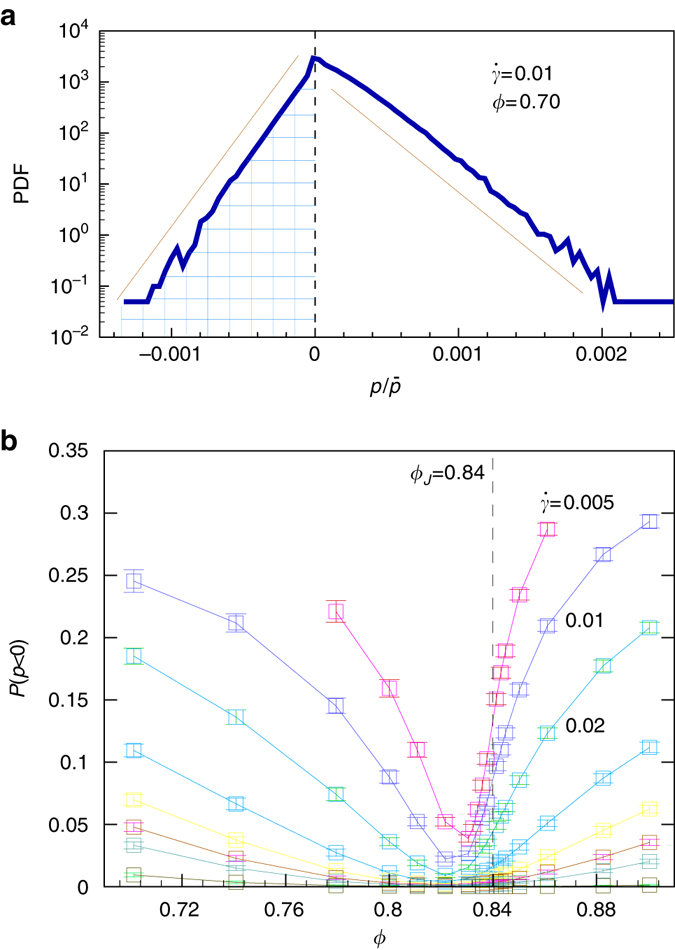
Probability of rare fluctuations. **a** A typical PDF of the rescaled power $p/\bar{p}$ for *ϕ* = 0.7, $\dot{\gamma }=0.01$, and *L* = 30. The *solid straight lines* show the exponential decay of the PDF for large and small arguments. The area of the shaded region gives the probability, *P*(*p* < 0), that the local power takes a negative value, i.e., to encounter a negative power injection. **b** The probability to observe a negative power injection *P*(*p* < 0) as a function of packing fraction *ϕ* for different shear rates $\dot{\gamma }=0.005,0.01,0.02,0.04,0.06,0.08,0.1$ and 0.2 from top to bottom, respectively, and system size *L* = 30. The vertical dashed line marks the critical packing fraction, *ϕ*_*J*_, where the jamming transition occurs in the static limit. Error bars correspond to square root of variance

In the Fig. 1a, we show a typical example of the PDF, ${\cal P}(p/{\bar p})$, of the local power flux rescaled by the mean power, i.e., $p/{\bar p}$. The PDF exhibits several remarkable features. The power flux can take negative values with a rather high probability. The distribution is strongly skewed towards positive events. At the both sides the PDF decays exponentially to a good approximations. It is very different from a Gaussian distribution. Still, the negative part of the PDF (the *shaded area* in Fig. 1a) decreases rapidly with system size. In the following this area will be denoted as $P(p < 0)\equiv {\int }_{-\infty }^{0}{\cal P}(p)\,{\rm d}p$. The slopes of the exponential decay are roughly proportional to the number of particles in the considered volume such that *P*(*p* < 0) decays exponentially to zero with system size.

**Fig. 2:**
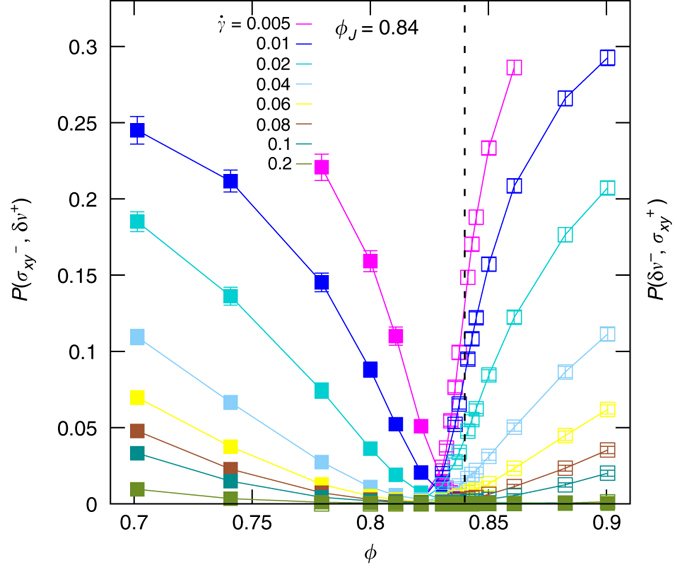
Mutually exclusive fluctuations. Joint probabilities $P({\sigma }_{xy}^{-},\delta {v}^{+})$ (left axis, filled symbols) and $P(\delta {v}^{-},\,{\sigma }_{xy}^{+})$ (right axis, hollow symbols) as functions of packing fraction, *ϕ*, for various shear rates, $\dot{\gamma }$. In the fluid state, *ϕ* < *ϕ*_*J*_, the dominant mechanism of negative power injection is the reversal of the shear stress. In the jammed state, *ϕ* > *ϕ*_*J*_, it is due to the reversion of the velocity gradient. Error bars correspond to square root of variance

**Fig. 3:**
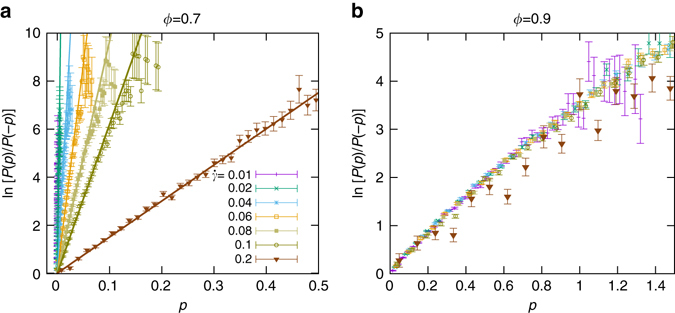
Verification of the instantaneous FR. Plot of $\rm {ln}\,[P(p)/P(-p)]$ vs. *p* for two packing fractions **a** *ϕ* = 0.7 and, **b** *ϕ* = 0.9. The *solid lines* are linear fits of slope *β*_*e*_ = *τ*/*T*_*e*_ of the data for different shear rates. The slope decreases by increasing the shear rate $\dot{\gamma }$, implying that the effective temperature *T*_*e*_ increases by $\dot{\gamma }$. The slope has a weak dependence on $\dot{\gamma }$ in the jammed state. For *n* ^+^ and *n* ^−^ representing number of positive (+*p*) and negative (−*p*) cases, the corresponding error bar of *P*(*p*)/*P*(−*p*) is equal to (1/*n* ^+^+1/*n* ^−^)^1/2^

**Fig. 4:**
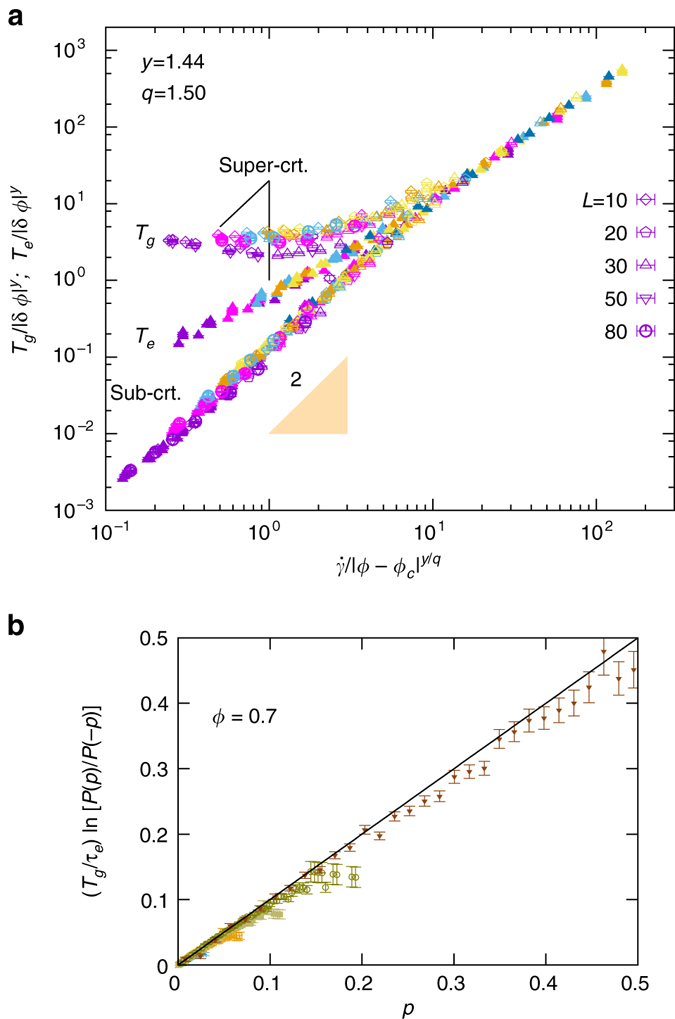
Scaling of effective and kinetic temperatures. **a** When rescaled with the critical exponents *q* = 1.5(1) and *y* = 1.44(15) the effective and granular temperatures collapse onto a scaling function. However, to achieve a data collapse we had to adopt slightly different critical densities, *ϕ*_*c*_ = 0.83 and *ϕ*_*c*_ = *ϕ*_*J*_ = 0.84 for *T*_*e*_ and *T*_*g*_, respectively. In the fluid state and in the critical state the temperatures match. For the fluid state they exhibit Bagnoldian scaling with exponent 2. In the critical state they still share same non-trivial scaling for $\dot{\gamma }/\delta {\phi }^{y/q}\gtrapprox 10$. In the jammed state the temperatures segregate into two different branches; *T*_*e*_ approaches a constant and *T*_*g*_ follows a power-law behavior with exponent 1.5(1). Different system sizes are given by different symbols in which filled and hollow symbols refer to *T*_*g*_ and *T*_*e*_, respectively. The color code corresponds to different shear rates $\dot{\gamma }=0.02$ (*purple*), 0.04 (*magenta*), 0.06 (*blue*), 0.08 (*golden*), and 0.1 (*yellow*). **b** The collapse of all data presented in Fig. 4a when the vertical axis is multiplied by a factor of *T*_*g*_/*τ* with *τ* = 0.28. In these data, we cover a large range of packing fractions around jamming, 0.7 < *ϕ* < 0.9. Error bars correspond to square root of variance

### Probability of rare fluctuations

In Fig. 1b we show *P*(*p* < 0) as a function of packing fraction *ϕ*. The *lines* in *different color* indicate data for different shear rates, ${\dot \gamma }$. For shear rates, ${\dot \gamma }\gtrapprox 0.2$ (olive), this probability is very small, and it grows upon decreasing the shear rate. There is pronounced growth in the fluid and in the jammed states. However, close to the jamming point *ϕ*_*J*_ (marked by the *vertical dashed line*) it remains small for all shear rates. The critical point lies to the right of the minima. However, the minima converge towards *ϕ*_*J*_ in the limit ${\dot \gamma }\to 0$ and system size *L*→∞. Hence, the minimum will eventually approach *ϕ*_*J*_; the discrepancy is due to finite-size effects. The emergence of the global minimum is remarkable because fluctuations are expected to diverge close to a critical point^16–18^, i.e., one expects a larger probability to encounter fluctuations close to the critical point. The minimum is a distinctive feature of the jamming transition. It has no counterpart in equilibrium thermodynamics.

### Decomposition of the probability of rare fluctuations

In order to understand the origins of the negative power injection, we recall that the power flux has two contributions: The local shear stress *σ*_*xy*_, and the local velocity gradient *δv*. Negative power injection arises whenever either *σ*_*xy*_ < 0 and *δv* > 0, or *δv* < 0 and *σ*_*xy*_ > 0. The former events will be denoted as $({\sigma }_{xy}^{-},\delta {v}^{+})$. In this case the velocity profile remains monotonic and negative power injection arises from fluctuations of the shear stress. The latter events will be denoted as $(\delta {v}^{-},{\sigma }_{xy}^{+})$. In this case the negative power injection is connected to rare fluctuations where the velocity profile is no longer monotonic (see Supplementary Fig. 1). The joint probability of these events sum up to the probability to encounter negative power injection: $P(p < 0)=P({\sigma }_{xy}^{-},\delta {v}^{+})+P(\delta {v}^{-},{\sigma }_{xy}^{+})$ (In Supplementary Note 5 and Supplementary Fig. 3 we numerically prove this equality). Figure 2 presents the numerical results for these two joint probabilities, i.e., $P({\sigma }_{xy}^{-},\delta {v}^{+})$ (left axis, *filled symbols*) and $P(\delta {v}^{-},{\sigma }_{xy}^{+})$ (right axis, *hollow symbols*) as function of packing fraction *ϕ* for various shear rates. For a given shear rate (a given color), the intersection point of the joint probabilities is very close to the jamming point. Hence, *ϕ*_*J*_ splits the probability space into two disjoint regions. Accordingly, the two types of mechanisms, shear-stress and velocity-gradient fluctuations, are mutually exclusive. The reason can be sketched as follows. The positive shear stress corresponds to head-to-head collisions while the negative shear stress is associated to backup collisions during which the local angular momentum is in the same and in the opposite direction of the global induced angular momentum of the flow, respectively (see Supplementary Fig. 2 and Supplementary Note 4). For *ϕ* < *ϕ*_*J*_, since the average coordination number is relatively small, the negative contacts can be observed with high probability although they are suppressed by increasing the shearing flow. This justifies the decreasing dependence of the probability of negative stress as function of both shear rate and packing fraction. For *ϕ* > *ϕ*_*J*_, since the coordination number jumps to a value ≥4, the negative contacts can in average be suppressed by the positive ones since the global symmetries of the flow favor the positive contacts. This elucidates the behavior seen in Fig. 2 for the negative shear stress and since the negative power includes exclusive contributions from the negative shear stress and negative velocity gradient, our argument naturally explains the observation of the mutually exclusive fluctuations.

### Fluctuation relation

At this point we identified qualitatively different physics underlying the fluctuations of fluid and jammed systems. In order to gain more insight into the parameter dependence of the strength of fluctuations we establish now a FR for the flows. It will characterize the width of the PDFs ${\cal P}(p)$ by an effective temperature *T*_*e*_. We conjecture that the relation $\rm{ln}\,[P(p)/P(-p)]=\beta p$ holds where *β* = *τ*/*T*_*e*_ has inverse dimension of power. Here, the constant *τ* is the relevant elastic time scale which represents the typical time scale of a single collision. It is approximately independent of *ϕ* and $\dot{\gamma }$ (see Supplementary Note 6).

Figure 3 presents the numerical verification of our conjecture for two packing fractions *ϕ* = 0.7 and 0.9 that lie below and above the critical point, *ϕ*_*J*_ = 0.84, respectively. There is a linear dependence between $\rm{ln}\,[P(p)/P(-p)]$ and *p* whose slope is a decreasing function of the shear rate $\dot{\gamma }$. This implies that *T*_*e*_ is an increasing function of the shear rate, in accordance with the shear-rate dependence of the average kinetic temperature of particles in the flow. Surprisingly, the correspondence is not only qualitative. It even holds quantitatively. All data shown in Fig. 3a collapse to a straight line when $\rm{ln}\,[P(p)/P(-p)]$ is multiplied by the granular temperature, *T*_*g*_ (Fig. 3b). The resulting straight line has a slope 1 with *τ* = 0.28. (In Supplementary Note 8 and Supplementary Fig. 8, we numerically prove that our proposed FR is also satisfied for highly damped systems corresponding to non-Brownian suspensions.)

### Scaling collapse of effective and kinetic temperatures

In the jammed state the effective temperature, *T*_*e*_, and the granular temperature, *T*_*g*_, differ: *T*_*e*_ is always larger than *T*_*g*_. We explore the parameter dependence of the two temperatures by exploring their scaling properties^19^. In Fig. 4a we demonstrate that the full $\dot{\gamma }$ and *ϕ* dependence of the temperatures for different system sizes can be represented in terms of a master plot where *T*/|*δϕ*|^*y*^ is plotted as function of $\dot{\gamma }/|\delta \phi {|}^{y/q}$ with appropriate choice of the exponents *y*, *q* and *δϕ* = *ϕ*−*ϕ*_*c*_. For the fluid state we thus find the well-known Bagnoldian scaling with exponent 2. In the jammed state, we find that *T*_*e*_ and *T*_*g*_ still collapse uniformly in the critical region, $\dot{\gamma }/|\delta \phi {|}^{y/q}\gtrapprox 10$. For jammed flows, however, they segregate into two different branches in accord with our earlier statement. In this limit *T*_*e*_ approaches a constant—yield stress emerges. In contrast, *T*_*g*_ shows a power-law behavior with exponent ∼ 1.5(1). We have checked consistency of all our exponents, i.e., exponents of granular temperature and components of stress tensor, in Supplementary Note 7 and Supplementary Figs 4–7. We also show in Supplementary Note 8 that these exponents are universal in a sense that the same scaling collapse is achieved with the same critical exponents for highly dissipative regime, in connection with the non-Brownian suspensions (See Supplementary Fig. 9).

Heussinger *et al*.^20, 21^ have studied fluctuations of some observables in the flow of an assembly of frictionless, soft discs at zero temperature, in the vicinity of and slightly above *ϕ*_*J*_. They have found that the contact-number fluctuations and relative fluctuations of the shear stress diverge upon approaching *ϕ*_*J*_ from above. They also report on strong finite-size effects when using *ϕ* as control parameter. However, the effective temperature *T*_*e*_ in our study, is the product of thermodynamically forbidden fluctuations (negative stress below *ϕ*_*J*_ and negative velocity gradient above *ϕ*_*J*_) which are specific to small-size systems and vanish in the vicinity of *ϕ*_*J*_. Our observation indicates that the scaling behavior of *T*_*e*_ is not significantly altered by the finite-size effects below and above *ϕ*_*J*_ for *L* > 10 (see Fig. 4a). We find that to a very good extent, *T*_*e*_ is independent of the system size. We only see small deviations for *L* = 10. These deviations vanish as $\dot{\gamma }\to 0$.

## Discussion

The flow of particulate matter is similar to classical fluids in so far as it involves the motion of many particles that interact by short-range forces. As function of packing fraction the flows undergo a phase transition from a fluid into a jammed state. Close to the critical point the materials obey scaling relations^22, 23^, reminiscent of critical phenomena. The data collapse of the granular temperature, i.e., the kinetic energy per degree of freedom, is shown here in Fig. 4a. In the fluid state and in the critical region this temperature agrees with an effective temperature that characterizes the probability to encounter different power injections.

The proposed effective temperature is sensitive to the inherent properties of the systems, and it potentially qualifies as the effective temperature that has been searched for recently with great urgency^24, 25^. The effective temperatures proposed in the past^26^ are based on fluctuation–dissipation relations, i.e., they assume linear response. Our study goes beyond linear response by introducing a FR in order to define a shear-rate-dependent effective temperature. This effective temperature is valid for packing fractions far from the jamming point, in contrast to the previous ones that are meaningful measure only near the transition point^26^.

Various types of fluctuation theorems have been extensively studied over the last two decades^13, 27–29^. Motivated by molecular dynamics simulations, Evans *et al*.^30^ proposed an empirical FR for entropy production rate in a two-dimensional sheared Lennard–Jones fluid. Later, this empirical relation was rigorously proved by Gallavotti and Cohen^31, 32^. This is now known as steady-state fluctuation theorem (SSFT). In a SSFT, the entropy production rate is time averaged over a single, randomly sampled interval of duration *τ*. In contrast, the transient fluctuation theorem (TFT) of Evans and Searles^33^ applies to a system that evolves from an initial equilibrium state to a nonequilibrium steady state. TFTs are different from SSFTs from a practical point of view. Whereas TFRs rely on ensemble averaging all starting from the same initial macro-state, SSFRs may be verified from steady-state evolution of a system over a sufficiently long time^34^. As we have already stressed out, properties of soft particulate flows are predominated by the rare fluctuations which result to intermittent behavior of these flows. It can be seen that FRs of type SSFTs are not suitable for soft particulate flows. The reason is that as a consequence of averaging process during the sampling time, the rare fluctuations can be washed out. Therefore, we use an instantaneous, time-independent FR to characterize strength of rare fluctuations. Whether our postulated FR would enjoy a rigorous treatment, will remain a theoretical challenge.

In addition, we have shown here that fluctuations in soft particulate flows differ essentially from those of classical fluids. First of all, they are very strong as demonstrated by the exponential decay of the PDFs of negative power injection (Fig. 1a) rather than the much faster decay of Gaussian distributions. Even for shear rates as large as $\dot{\gamma }=0.2$ we observe negative power injection (lowermost curve in Fig. 1b). Even more surprising, rare fluctuations are strongly suppressed close to the critical point (minima of the curves in Fig. 1b). They behave exactly contrary to the strength of critical fluctuations that diverge at the critical point and die out rapidly outside the critical region^16^. It will be challenging numerically, but extremely interesting from a conceptual point of view to explore how classical fluids behave in this respect. Finally, we have shown that there are different physical mechanisms underlying rare fluctuations in fluid and jammed states: In fluid states negative power injection originates from fluctuations where the shear stress takes a negative sign, in jammed states they arise in regions with negative shear rates. This dichonometry of mutually exclusive mechanisms of rare fluctuations above and below a critical state is a distinctive feature of soft particulate flows that has no counterpart in equilibrium thermodynamics.

Rheological properties of particulate flows are commonly characterized in terms of hydrodynamic equations and constitutive relations^35, 36^. Fluctuations are not a part of the modeling. The present study takes a different approach to characterize the systems: We focus on the fluctuations as an inherent property of the dynamics. Remarkably, the fluctuations obey a local, time-independent FR, and this relation can be used to define an effective temperature of the system. In contrast to the hydrodynamic approaches the temperature is not a field variable in this setting. Rather it characterizes the variability of snapshots of the flow. It is a scalar quantity that characterizes the ensemble of observations of the flows, taking full note of fluctuations. It neither requires to find appropriate heuristic constitutive equations, nor does it rely on the scale separation at mesoscopic scales that is implicit to the definition of thermodynamic fields. Hence, it is less prone to pitfalls arising from inapt choices of constitutive relations and applicable to a larger class of far-from-equilibrium flows. Thus, the present study opens a qualitatively new road to the description of far-from-equilibrium particulate flows.

In a recent study, Maloney^37^ investigated distribution of dissipation power ${\cal{P}}(\Gamma )$ in Durian’s bubble model. Whereas we find that ${\cal{P}}(p)$ has always exponential tails, it is shown that above jamming density ${\cal{P}}(\Gamma )$ becomes power law for small shear rates. This implies that distributions of injection and dissipation powers might not be equivalent. Stationarity condition implies that first moments of these distributions must be equal. But as one can see, higher moments of these distributions, which refer to the characteristic of the tails, might be different. This suggests a new avenue of research for investigation of steady state properties of non-equilibrium states. In this context, Maloney’s work^37^ together with our approach to calculate ${\cal{P}}(p)$ in shear flows provide a solid framework for investigation of similarities and differences between distributions of injection and dissipation powers.

## Methods

### Simulation details

We perform molecular-dynamics simulations of two-dimensional frictionless bidisperse disks. Particles interact via short range repulsive and dissipative forces. Two particles *i* and *j* of radii ${R}_{i}^{a}$ and ${R}_{j}^{b}$ (where *a*, *b* = 0, 1 stand for two different radius of bidisperse particles) at positions **r**_*i*_ and **r**_*j*_ interact when ${\xi }_{ij}={R}_{i}^{a}+{R}_{j}^{b}-{r}_{ij} >0$. Here *ξ*_*ij*_ is called the mutual compression of particles *i* and *j*, *r*_*ij*_ = |**r**_*i*_−**r**_*j*_|. The particles interact via a linear Dashpot model, *F*_*ij*_ = *Yξ*_*ij*_+*γ* ${{\rm d}\xi_{ij}\over {\rm d}t}$, where *Y* and *γ* are denoted as elastic and dissipative constant, respectively. Throughout the study we adopt the values *Y* = 100 and *γ* = 0.315, respectively.

In order to prevent crystallization we use a 1:1 binary mixture of particles where the ratio of the radii of large and small particles is set to *R* ^1^/*R* ^0^ = 1.4.

The equations of motion are non-dimensionalized by choosing the unit of the length to be *R* ^0^+*R* ^1^ = 1, and setting the mass of each particle equal to its area, *m*_*a*_ = *π*[*R* ^*a*^]^2^. Finally, the ratio of *Y* and *γ* provides the time scale ${t}^{\star }=\gamma /Y=3.15\times {10}^{-3}$.

Lees–Edwards boundary conditions are applied along *x*-direction. They create a uniform overall shear rate, $\dot{\gamma }$. These equations of motion are integrated with a 5th-order predictor-corrector Gear algorithm with time step, d*t* = 10^−4^.

### Data availability

The authors declare that the data supporting the findings of this study are available from the authors on request.

## Electronic Supplementary Material

Supplementary Information Supplementary Figures, Supplementary Notes and Supplementary References

Peer Review File Reviewer reports and authors' response from the peer review of this Article at Nature Communications
