# Supplementary material for: Characterizing rare fluctuations in soft particulate flows
Source: Nat Commun. 2017 Apr 10;8:11. doi: 10.1038/s41467-017-00022-8 (PMC5431914; doi:10.1038/s41467-017-00022-8)
Supplement: Supplementary file 1 — Supplementary Figures, Supplementary Notes and Supplementary References [file 41467_2017_22_MOESM1_ESM.pdf]

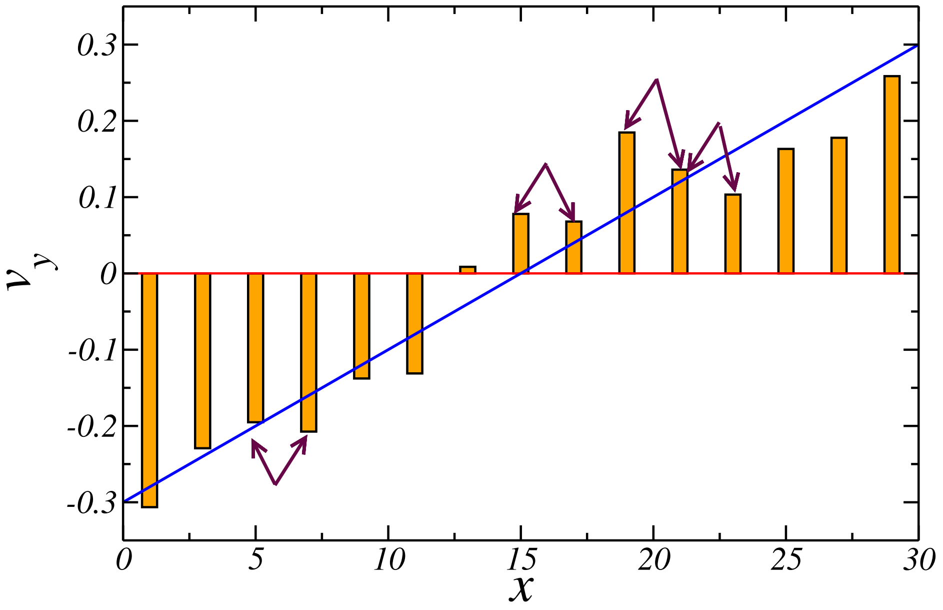

Supplementary Figure 1. **Negative Shear velocity**. The blue line shows the linear profile given by Supplementary Eq.13 which is recovered, for both $\phi < \phi_J$ and $\phi > \phi_J$, after a time averaging is done over different snapshots of the velocity profile. The golden bars show an instantaneous local drift velocity. One can see that the instantaneous velocity profile is non-monotonic and deviates from the averaged linear profile at each point. Furthermore, at four points, marked by the maroon arrows, the velocity gradient becomes negative, i.e., $\delta v_i < 0$. We refer to this as negative shear velocity or negative velocity gradient. In this simulation the system size is $L = 30$, the packing fraction is $\phi = 0.9$, and the shear rate is $\dot{\gamma} = 0.02$.

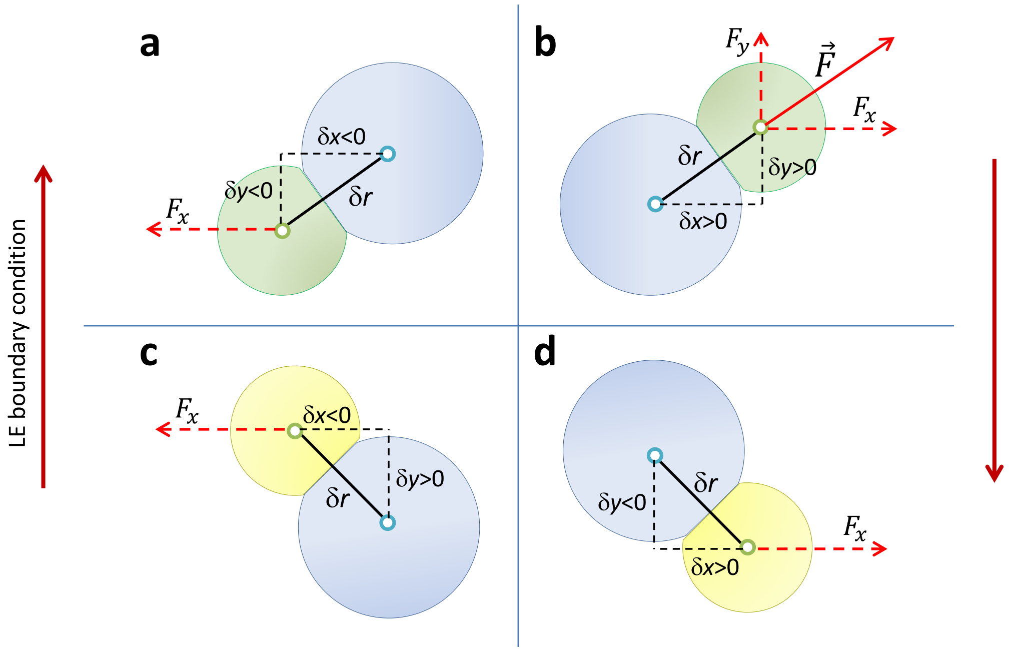

Supplementary Figure 2. **Negative Shear stress**. Long force chains are built up by segments of the interacting particles. Different orientation of local force chains in the particle level are shown. The shearing favors cases **a** and **b** where the contribution of the colliding particles to the shear stress is positive (positive orientation). These cases correspond to head-to-head collisions. However, cases **c** and **d** correspond to negative contribution to the shear stress (negative orientation). These cases correspond to backup collisions where colliding particles move against the flow. It is clear that due to the shearing, positive orientations occur much more frequently than the negative orientations. This is why the negative orientations (backup collisions) are rare events.

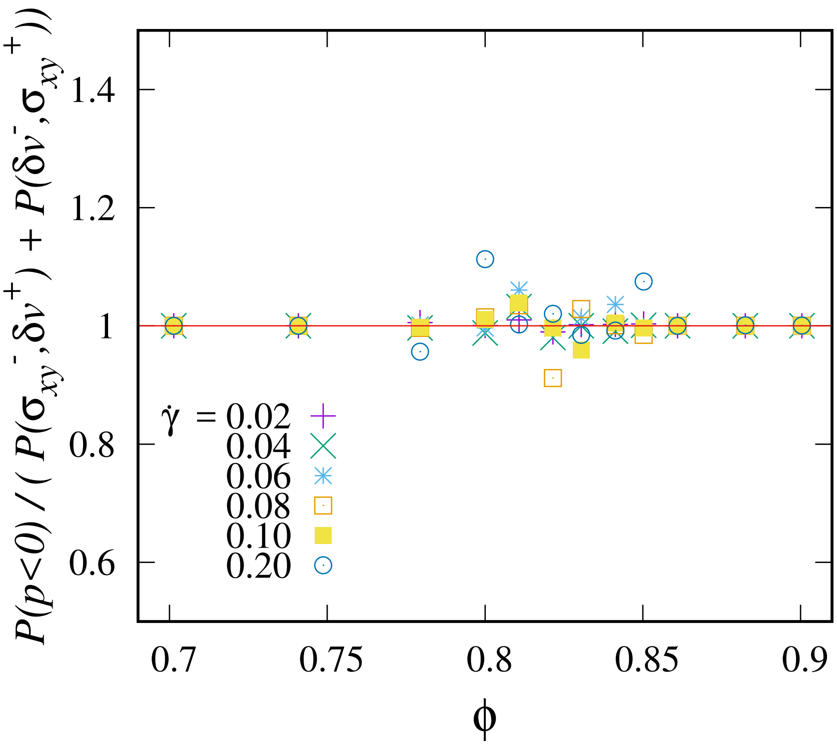

Supplementary Figure 3. **Balance of probabilities.** Here we verify that ratio of negative power fluxes, $P(p<0)$, to sum of the joint probabilities of the reverse shear forces, $P(\sigma_{xy}^{-}, \delta v^{+})$, and the reverse shear velocities, $P(\delta v^{-}, \sigma_{xy}^{+})$, equals to unity.

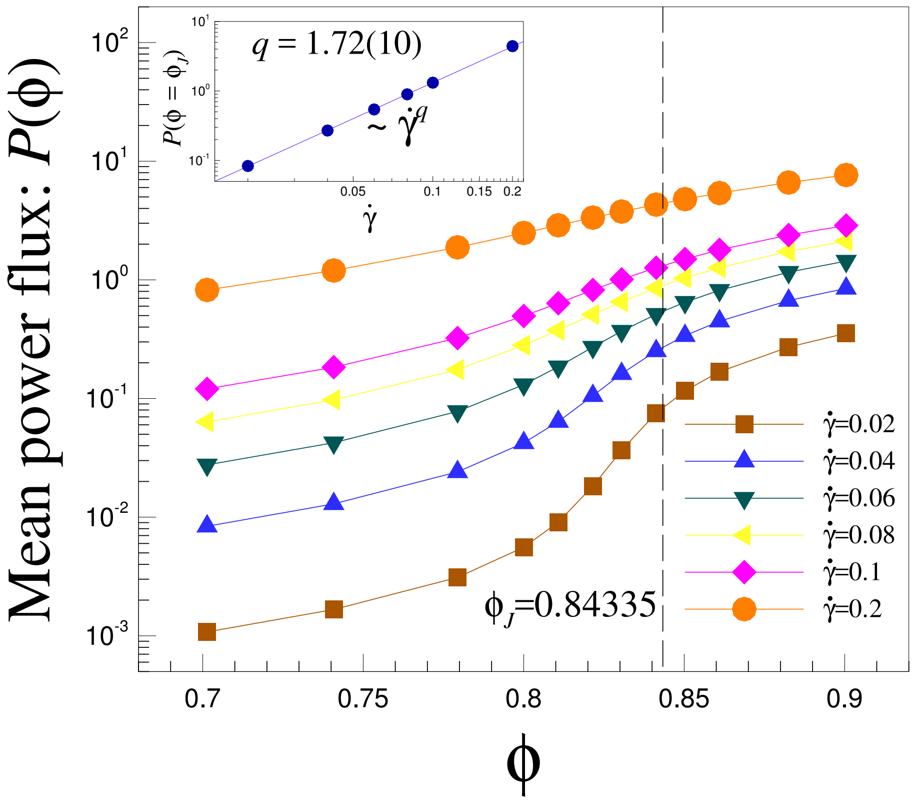

Supplementary Figure 4. **Mean injection power.** Main: Mean power flux $P(\phi)$ in a system of linear size $L = 30$ as a function of packing fraction ϕ for different shear rates $\dot{\gamma} = 0.02, 0.04, 0.06, 0.08, 0.1$ and 0.2. The vertical dashed line shows the critical packing fraction ϕ_J at which the jamming transition occurs. Inset: Scaling behavior of the mean power flux at the jamming transition point as a function of shear rate, gives the critical exponent $q = 1.72(10)$.

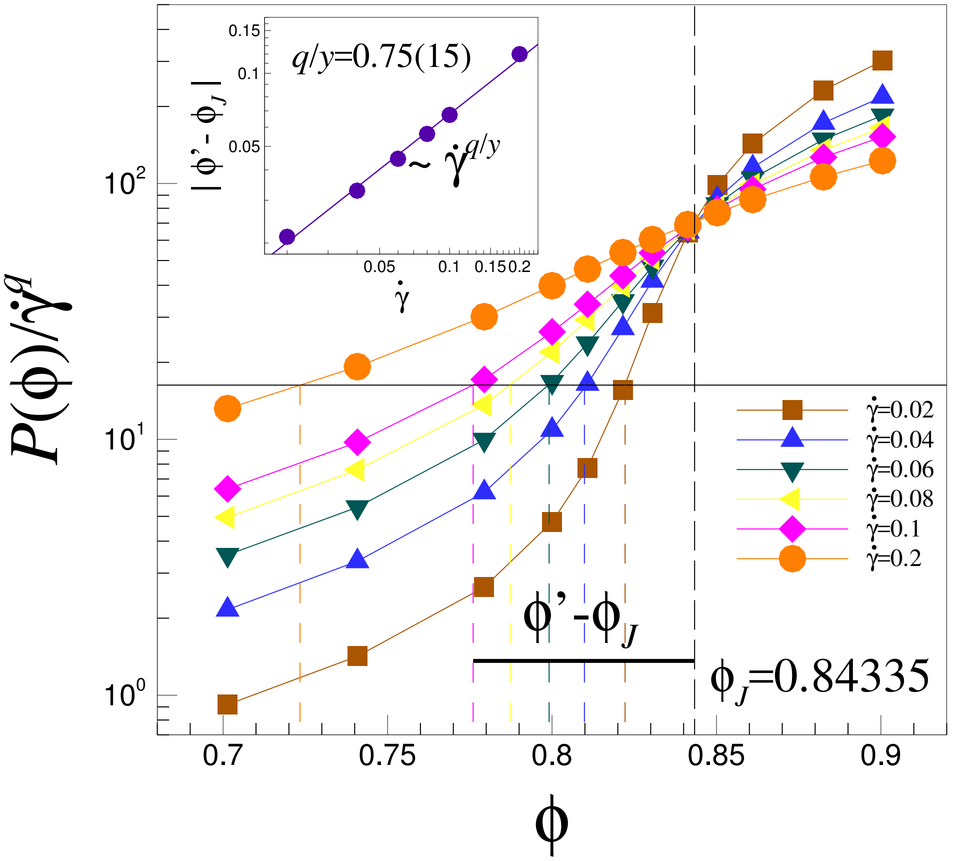

Supplementary Figure 5. **Semi-scaled mean injection power.** Main: Rescaling of the data along the y-axis in terms of the scaling exponent q obtained in Supplementary Fig. 4 at ϕ_J. Inset: Scaling behavior of the distance between the intersection ϕ' of the horizontal solid line with each curve and the vertical dashed line passing through ϕ_J.

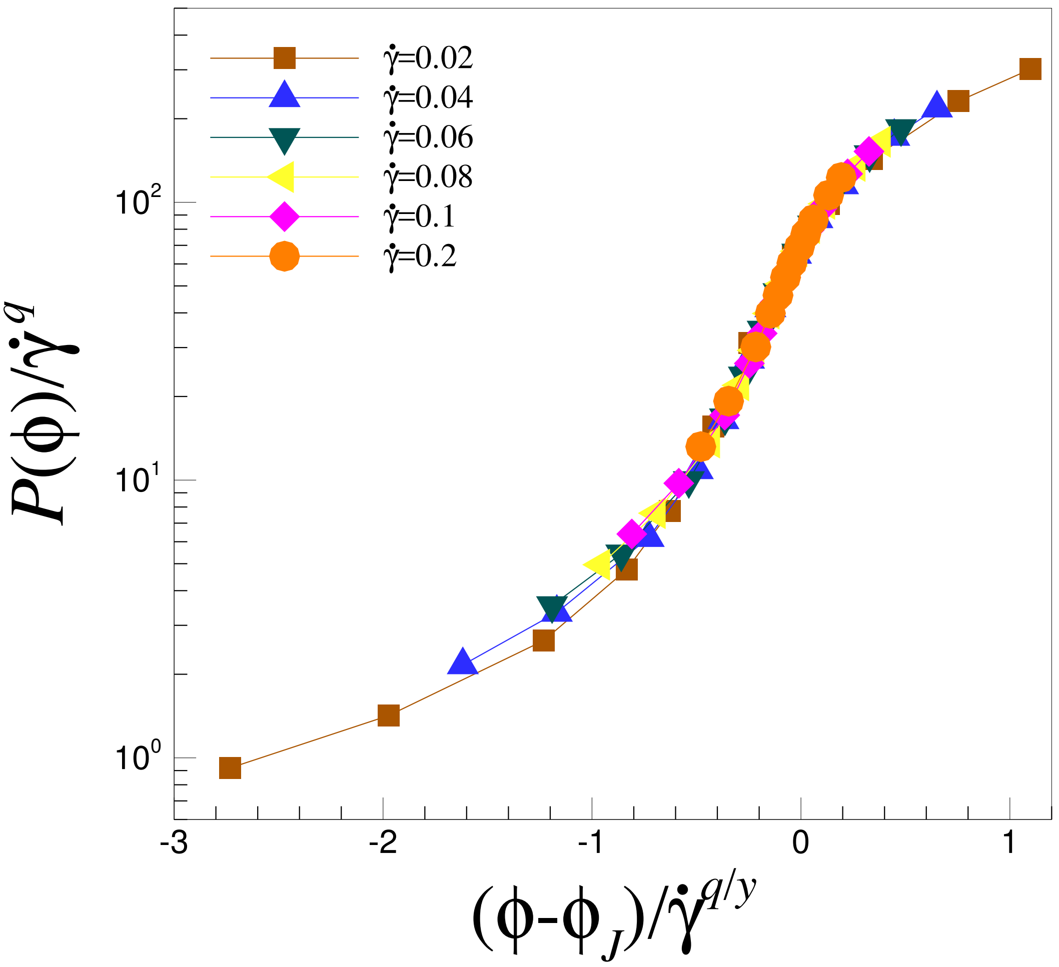

Supplementary Figure 6. **Collapse of mean power for $|\delta\phi| \leq 10^{-1}$.** Data collapse for the mean power flux by using the exponents obtained in Supplementary Figs. 4 and 5.

.

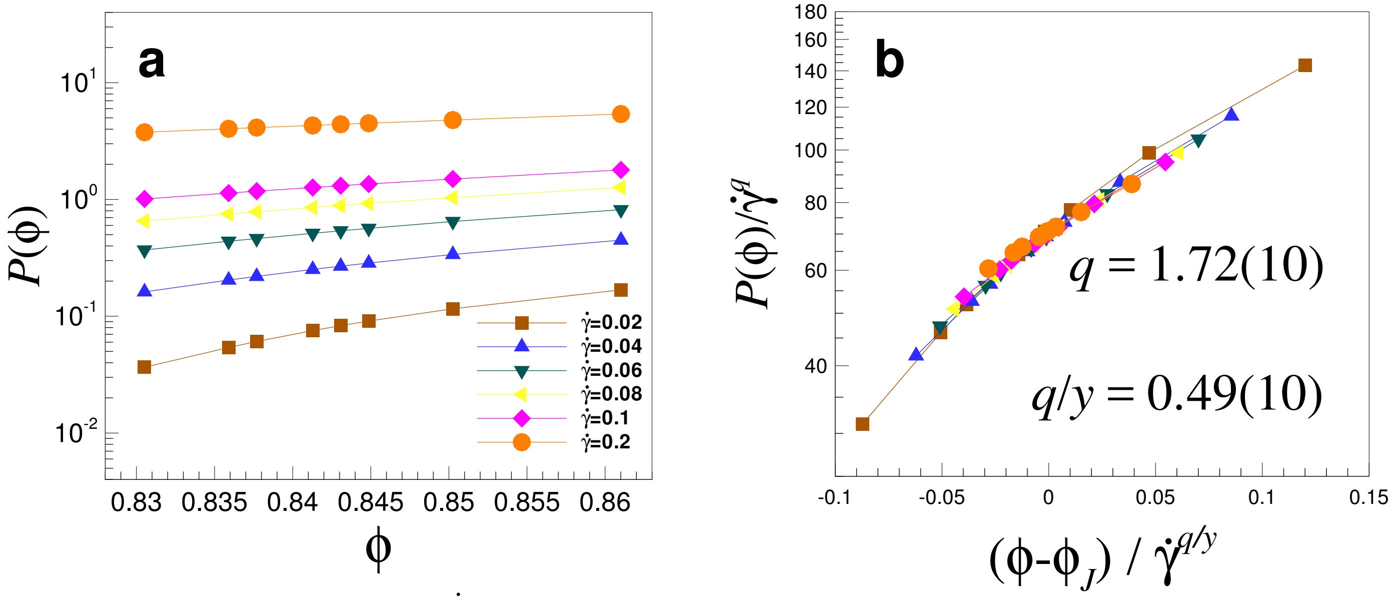

Supplementary Figure 7. **Collapse of mean power for $|\delta\phi| \leq 10^{-2}$.** Data collapse (**b**) for the mean power flux (**a**) very close to the jamming transition point with modified exponents in close agreement with those reported by Hatano [6].

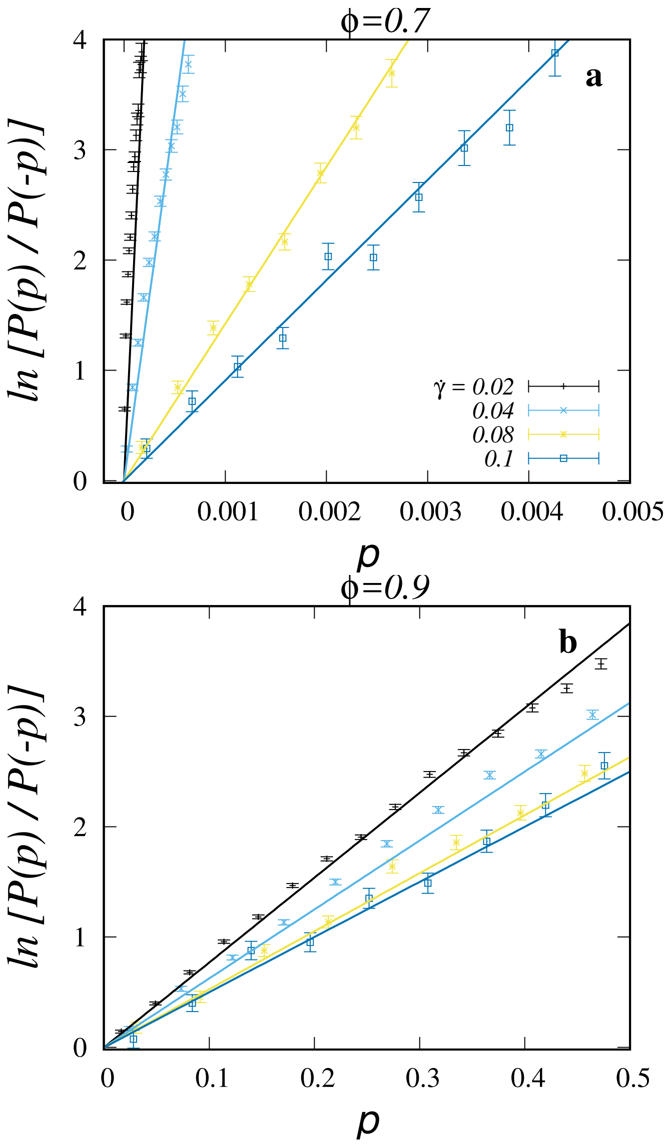

Supplementary Figure 8. **Verification of FT for highly dissipative system.** Plot of $\ln[P(p)/P(-p)]$ vs p for two packing fractions $\phi = 0.7$ (left) and $\phi = 0.9$ (right). The damping coefficient is $\gamma = 8.95$ and the spring prefactor is $Y = 100$. This corresponds to a highly dissipative system. The solid lines are linear fits of slope $\beta_e = \tau/T_e$ to the data for different shear rates where T_e is the effective temperature and τ is the refined time scale. The slope decreases by increasing the shear rate $\dot{\gamma}$, implying that the effective temperature T_e increases by $\dot{\gamma}$. The slope has a weak dependence on $\dot{\gamma}$ in the jammed state. For n^+ and n^- representing number of positive $(+p)$ and negative $(-p)$ cases, the corresponding error bar of $P(p)/P(-p)$ is equal to $(1/n^+ + 1/n^-)^{1/2}$.

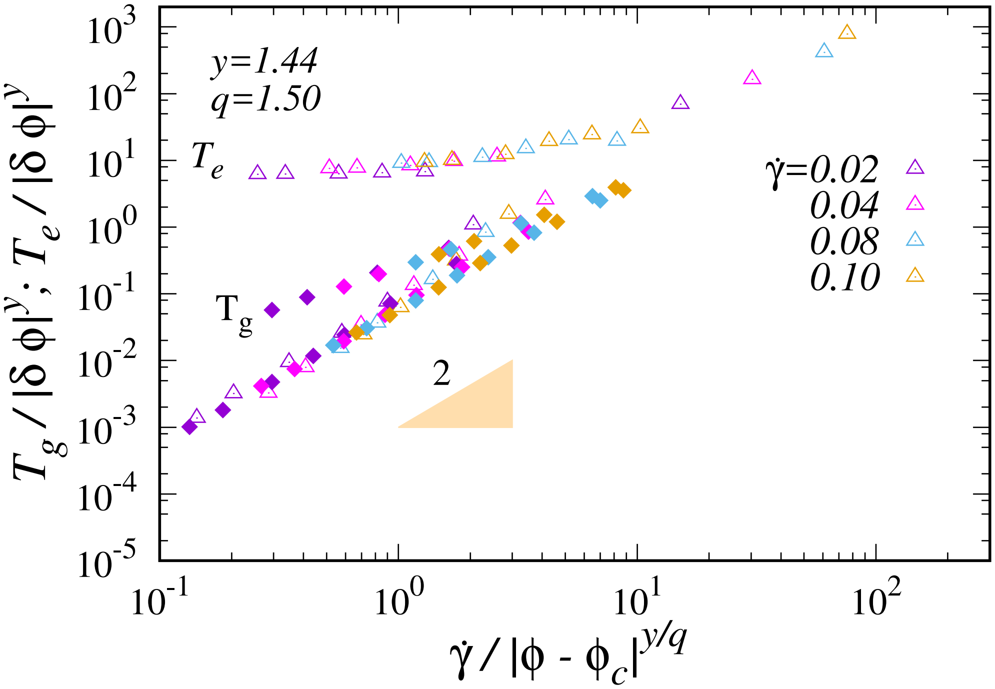

Supplementary Figure 9. **Scaling collapse of T_e and T_g for highly dissipative system.** $T_g/|\delta\phi|^y$ and $T_e/|\delta\phi|^y$ vs $\dot{\gamma}/|\phi - \phi_c|^{y/q}$ are plotted. Filled symbols refer to T_g and hollow symbols correspond to T_e. The color code shows different shear rates. Here, we adopt exactly the same exponents and critical densities as we used for the quasi-elastic systems. This demonstrates the robustness of universality against changes in the dissipation in the system. The damping coefficient is $\gamma = 8.95$ and the spring constant is $Y = 100$.

Supplementary Note 1. COMPUTATIONS AND MEASUREMENTS

A. Numerical Constants

Numerical constants are as follows: i) the time step is $dt = 10^{-4}$, ii) the elastic and dissipative constants for the linear Dashpot model are $Y = 100$ and $\gamma = 0.315$, respectively. The latter constants, give rise to a time scale $t^\star = \gamma/Y = 3.15 \times 10^{-3}$ which can be used to non-dimensionalize other quantities.

B. Artificial attractive force and coefficient of restitution

As a consequence of the dissipation in the linear Dashpot model, absolute value of the post-collisional normal relative velocity, g', will be smaller than the pre-collisional value, g, i.e., $g' < g$. Coefficient of the restitution is defined as $\epsilon = g'/g$ which is then in the range $0 < \epsilon < 1$ where 0 and 1 correspond to fully dissipative and elastic collisions, respectively. By integrating Newton's equation of motion, the coefficient of the restitution, ϵ, can be easily calculated

$$\epsilon = \exp(-\frac{\pi\gamma}{2m_{eff}}/\sqrt{\frac{Y}{m_{eff}} - \left(\frac{\gamma}{2m_{eff}}\right)^2}) \tag{1}$$

where $m_{eff} = m_i m_j/(m_i + m_j)$ is the effective mass of colliding particles. Inspection of Supplementary Eq. 1 indicates that in the linear Dashpot model the coefficient of restitution is independent of the impact velocities. This model has been widely used for comparison of the numerical results with that obtained from continuum mechanics and kinetic theory of granular systems.

From Supplementary Eq. 1, the restitution coefficient in our study (according to the adopted values reported in Supplementary Note 1 A) is $\epsilon = 0.92$ which is a typical value for granular systems. The linear Dashpot model occasionally gives rise to an artificial attractive force. To prevent this, we enforce zero force between colliding particles when the net force becomes negative. This modification increases the restitution coefficient to $\epsilon' \simeq 0.96$ [1].

C. Measurements

Simulations are done in a large range of parameters. The packing fraction is considered in the range $0.7 < \phi < 0.9$ where the jamming is $\phi_J = 0.84$ [2]. The shear rate is in the range $0.02 < \dot{\gamma} < 0.2$. Dimensionless shear rate, $\dot{\gamma}^\star$ can be defined as $\dot{\gamma}^\star = t^\star \times \dot{\gamma} = \dot{\gamma} \times \gamma/Y$. Therefore, the dimensionless shear rate will be in the range $10^{-5} \lesssim \dot{\gamma}^\star \lesssim 10^{-4}$. For the finite size scaling analysis, we adopt different system sizes; $L = 10$, 20, 30, and 50. As a result, number of particles varies between 100 to 3000.

Rather than fixing the total time for simulations with different shear rates, we fix the total strain to $35 \times L$ where L is the box size. As a consequence, the total simulation time increases for smaller values of shear rate. We start to measure physical quantities when $5 \times L$ strain is reached. This limit ensures balance between the injection of energy and dissipation due to the Dashpot model.

The system is divided into narrow rectangular bins of area $2 \times L$ along the flow direction and thus $L/2$ stripes in each simulation box. The aim of this is to measure relevant physical quantities locally as function of the spatial position of the bin. Due to the Lees-Edwards boundary condition imposed along the x-direction, the average velocity profile depends only on x, and thus for a given bin at each position x, all local averages for the quantities of interest are taken over the particles in the y direction. During each simulation, we take 350 snapshots of all local physical quantities inside the bins. Quantities of interest are the shear stress σ_{xy}, the local drift velocity $v_y(x)$, and the granular temperature T_g (definition of these quantities are given in the next section).

We run 100 different realizations for each set of parameters. In the main paper, the averaged quantities such as shear stress σ_{xy}, and the injected-power p, are thus taken over 100×350 snapshots; each of which consists of $L/2$ entries for different bins. For example, for $L = 50$, we get 875×10^3 data points. To probe the aforementioned range of parameters, we performed altogether 20000 individual simulations; and on average one day of CPU-time for each simulation.

Supplementary Note 2. RELEVANT PHYSICAL QUANTITIES

Let us first define the shear stress σ_{xy} which is often measured and used in our calculations. At the spatial position x, the local shear stress $\sigma_{xy}(x)$ is calculated according to

$$\sigma_{xy}(x) = \frac{1}{A}\sum_{i}\frac{1}{2}m_i v_{i,x} \times (v_{i,y} - v_y(x)) + \frac{1}{A}\sum_{i<j} r_{ij,x} \times F_{ij,y}, \tag{2}$$

where $A = 2 \cdot L$ is the area of of the rectangular bin at position x, $v_{i,x}$ and $v_{i,y}$ are the x and y components of the velocity of the ith particle, respectively, $v_y(x)$ is the local drift velocity at x, $r_{ij,x}$ is the x component of the vector which connects the center of particle i to that of particle j, and $F_{ij,y}$ is the y component of the vector force exerted by particle i to j. We only consider the spring (potential) force in the calculation of the stress tensor. The first sum in the right hand side of Supplementary Eq. (2) runs through all particles at spatial position x, and the second sum runs over all neighboring particles that interact with particle i. If the neighboring particle belongs to a neighboring bin, we only consider half of the second term for each bin. With this local shear stress, it is straightforward to calculate the global shear stress Σ_{xy} in the system:

$$\Sigma_{xy} = \frac{1}{L}\int_0^L \sigma_{xy}(x)dx. \tag{3}$$

Granular temperature T_g is defined as the random kinetic energy of particles. Here, the randomness means that the drift velocity is subtracted from velocity of each particle, so that the temperature can be defined as:

$$T_g = \frac{1}{N}\sum_{i=1}^{N}\frac{1}{2}m_i\left(\langle(v_{i,y} - v_y(x))^2\rangle\right) \tag{4}$$

where the sum over i runs over N number of particles.

Supplementary Note 3. INJECTED POWER

A. Global injected power

In Lees-Edwards Boundary Conditions (LEBC) the energy injected into the system is defined as

$$I = \overline{\Sigma}_{xy} \times \dot{\gamma} \times L^2 \tag{5}$$

where $\overline{\Sigma}_{xy}$ is the time averaged global shear stress in the system, $\dot{\gamma}$ is the shear rate, and L is the system size. Whereas the global shear stress is a fluctuating quantity, the global shear rate $\dot{\gamma}$ is fixed by the boundary conditions and does not fluctuate. On average, the total injected power should be compensated by the dissipation power due to the linear Dashpot model. We have numerically checked the ratio of the injected and dissipated powers up to 10^{-3} precision for a wide range of shear rates and packing fraction and verified that: the flow reaches a non-equilibrium stationary state where the injected power is compensated by the dissipation and, as a result, Supplementary Eq. (2) faithfully describes the local shear stress. Furthermore, the former condition, i.e., balance between dissipation and injection, is used as a cross-check throughout all simulations as the stationarity condition of shearing.

B. Local injected power

Using Supplementary Eq.5, it is straightforward to calculate the local injected power which is the main physical quantity investigated in our study. We consider the space between centers of two neighboring bins as a thermodynamic subsystem. Each bin is identified by an integer number i, which runs from 0 (the most left bin), to $L/2-1$ (the most right bin). Then the local power flux in $2D$ can be defined as

$$p_i = \overline{\sigma}_{xy}^{(i)} \times L \times \delta v_i, \tag{6}$$

with

$$\overline{\sigma}_{xy}^{(i)} = \frac{\sigma_{xy}^{(i)} + \sigma_{xy}^{(i+1)}}{2}, \tag{7}$$

where $\sigma_{xy}^{(i)}$ and $\sigma_{xy}^{(i+1)}$ are the respective local shear stress of bins i and $i+1$ and $\overline{\sigma}_{xy}^{(i)}$ is the average local shear stress in the bins i and $i+1$, and $\delta v_i = v_{i+1} - v_i$ is the local shear velocity gradient.

Time averaged sum over local power fluxes in the system must be equal to the global injected power given by Supplementary Eq.5. To see this, we start by summing up the local power over all bins

$$\langle \sum_{i=0}^{L/2-1} p_i \rangle = \langle \sum_{i=0}^{L/2-1} \overline{\sigma}_{xy}^{(i)} \times L \times \delta v_i \rangle \tag{8}$$

where $\langle , \rangle$ denotes the time averaging. Since we have already shown that velocity gradient and shear stress are mutually exclusive, then to a good extent we can write $\langle \overline{\sigma}_{xy}^{(i)} \times \delta v_i \rangle = \langle \overline{\sigma}_{xy}^{(i)} \rangle \langle \delta v_i \rangle$. Using the fact that the time averaged mean shear stress is independent of the spatial position, we get:

$$\langle \sum_{i=0}^{L/2-1} p_i \rangle = \langle \overline{\sigma}_{xy} \rangle \times \sum_{i=0}^{L/2-1} L \times \langle \delta v_i \rangle \tag{9}$$

Because of the linear velocity profile, the time averaged mean velocity gradient is also the same for each point

$$\langle \sum_{i=0}^{L/2-1} p_i \rangle = \langle \overline{\sigma}_{xy} \rangle \times \langle \delta v \rangle \sum_{i=0}^{L/2-1} L \tag{10}$$

or

$$\langle \sum_{i=0}^{L/2-1} p_i \rangle = \langle \overline{\sigma}_{xy} \rangle \times \langle \delta v \rangle \times \frac{L^2}{2} \tag{11}$$

Since the distance between two neighboring bins is equal to 2, therefore $\langle \delta v \rangle / 2$ is equals to the global shear rate, $\dot{\gamma}$, as a results

$$\langle \sum_{i=0}^{L/2-1} p_i \rangle = \langle \overline{\sigma}_{xy} \rangle \times \dot{\gamma} \times L^2 \tag{12}$$

which is equivalent to Supplementary Eq.5. Note that, this definition of the forward and reverse flows is invariant under the Galilean transformation of the velocity profile which guarantees robustness of their definition.

One of the main messages of our study is based on the observation that the local power is a fluctuating quantity which can take both positive and negative values. Specifically, (**i**) for the positive case $p_i = p_i^+ > 0$ the subsystem gains a power flux equal to $p_i^+ = +|\overline{\sigma}_{xy}^{(i)} \times L \times \delta v_i|$ and (**ii**) for the negative case $p_i = p_i^- < 0$ the subsystem gives off a power flux equal to $p_i^- = -|\overline{\sigma}_{xy}^{(i)} \times L \times \delta v_i|$.

In analogy to the froward and reverse stochastic processes here the reverse and forward flows can be defined based on the following simple criterion:

$$\begin{cases} 1) \text{ Forward flow } \; p_i > 0; \\ 2) \text{ Reverse flow } \; p_i < 0. \end{cases}$$

Supplementary Note 4. ORIGIN OF THE NEGATIVE POWER

According to Supplementary Eq.6, a reverse flow $-p$ can be either caused by the reversal of the shear force or the reversal of the shear velocity. In order to clarify the origin of negative power in the preceding sections, we illustrate cases where either of these quantities become negative.

A. Negative shear velocities

In LEBCs, the velocity profile $v_y(x)$ attains a trivial linear function

$$v_y(x) = \dot{\gamma} \times (x - \frac{L}{2}) \tag{13}$$

where $\dot{\gamma}$ is the shear rate defined as the boundary velocity v_B divided by the system size L and x is the spatial coordinate. Supplementary Eq. 13 is only recovered when instantaneous velocity profiles over different times are averaged. However, instantaneous profiles possess fluctuations. For the time averaged profile, the difference of the drift velocity, δv_i, between two subsequent bins, i, and, $i+1$, is simply

$$\delta v_i = v_{i+1} - v_i \simeq d_{bin} \times \dot{\gamma} > 0 \tag{14}$$

where $d_{bin} = 2$ is the distance between centers of two successive bins. From Supplementary Eq. (13), it is clear that in a linear regime δv_i is strictly positive for any arbitrary bin number i. However, when the shear stress is localized somewhere, the difference of the drift velocity around such position can deviate from the one given by Supplementary Eq. (14) and may even become negative, i.e., $\delta v_i < 0$. We refer to the latter as negative shear velocity or negative velocity gradient. Whereas after averaging over different snapshots we can recover a linear profile given by Supplementary Eq. 13, we observe huge deviations from the linear profile for any instantaneous snapshot. Supplementary Fig. 1 shows the local drift velocity $v_y(x)$ as function of the spatial position x. The solid blue line shows the averaged linear velocity profile. However, one can see that the instantaneous velocity profile (the golden bars) deviates dramatically from the linear dependence. Moreover, we find that difference of the drift velocity between neighboring bins can also be negative, i.e., $\delta v_i < 0$. Examples of the negative shear velocities are marked by the maroon arrows. The packing fraction for this snapshot is $\phi = 0.9$. For $\phi > \phi_J$, flow profiles are non-monotonic

which is the main reason for negative velocity gradients. However, for $\phi < \phi_J$, although instantaneous velocity profiles have some fluctuations, the flow profiles respect monotonicity.

To investigate the origin of the negative shear velocities let us consider the following: If distribution functions of the drift velocities (v_y) inside neighboring bins overlap, then the reversal of the shear velocity will be a trivial consequence of the overlapping PDFs. To investigate this, we introduce a characteristic quantity as $\alpha = \langle (v_y - v_y(x))^2 \rangle^{1/2} / d_{bin}\dot{\gamma}$ which is ratio of the width of the distribution function of the drift velocities and difference of the velocity drift between two neighboring bins. If $\alpha \simeq 1$ then the negative velocity gradients are trivial. However for $\alpha << 1$ probability of overtaking from PDF of the neighboring bin will be negligible and these events will be rather due to interesting physics. To get insights about the nature of the negative shear velocities, within a good approximation one can suppose that distribution of velocities inside the bins are Gaussian; $P(v_y) \propto e^{-(v_y - v_y(x))^2/2\sigma^2}$ where $\sigma^2 = \langle (v_y - v_y(x))^2 \rangle = 2T_g/\overline{m}$, and $\overline{m} = \pi \times 0.5^2$. Therefore, $\alpha \approx T_g/\dot{\gamma}$. According to our computations, we find that for $\phi >> \phi_J$, $T_g = 0.22\dot{\gamma}$. Therefore, for that region $\alpha \approx 0.3$. Furthermore, for $\phi \approx \phi_J$, $T_g = 0.32\dot{\gamma}^{1.5}$ which accordingly $\alpha \approx 0.32\sqrt{\dot{\gamma}}$ which for the probed range of the shear rates we obtain $10^{-3} < \alpha < 10^{-2}$. Also for $\phi << \phi_J$, $T_g \propto \dot{\gamma}^2$, and thus $10^{-4} < \alpha < 10^{-3}$. Therefore, one can see that for all packing fractions we find $\alpha < 1$, and thus the negative shear velocity events are not trivially due to the overlap of the PDFs of the drift velocities of the particles.

B. Negative shear stress

The shear stress consists of two independent terms i) kinetic and ii) static (the first and second terms on the rhs of Supplementary Eq. 2). In the range of the shear rates in our study, the kinetic term is an order of magnitude smaller than the static term. Therefore, we only focus on the behavior of the static term. A particle can contribute in the static stress tensor only when it interacts with at least one of its neighbors. A typical example is given in Supplementary Fig. 2 where two colliding particles at distance δr are depicted. The force between two particles, F, is given by the linear Dashpot model with components $F_x = F\cos\theta$ and $F_y = F\sin\theta$ where $\cos\theta = \delta x/\delta r$ and $\sin\theta = \delta y/\delta r$. The contributing

term to the shear stress is given by

$$F_x \cdot \delta y = F \cdot \cos\theta \cdot \delta y = F \cdot \frac{\delta x \cdot \delta y}{\delta r} \quad (15)$$

In the above equation, strength of the force, F, and distance between particles, dr, are both strictly positive. Therefore, the only term which can become negative is $\delta x \cdot \delta y$. We show in Supplementary Fig. 2 that it is indeed the local orientation of force chains which can result to fluctuations in the sign of the stress tensor. Specifically, when the neighboring particle is either at first or third quadrants (cases **a** and **b** in Supplementary Fig. 2), Supplementary Eq.15 gives rise to a positive contribution. However, for the second or fourth quadrants (cases **c** and **d** in Supplementary Fig. 2) a negative contribution to the shear stress is obtained. We shall call the former and the latter as the positive and negative orientations, respectively. As a result, a negative stress is related to the local orientations of force chains in the system.

Now the question is that why physics of the system depends on the local orientation of the force chains. One should note that the symmetry of the system is broken by the applied external shearing. A positive orientation corresponds to a head-to-head collision which is naturally caused by the shearing. However, the negative orientation can only occur in the case of a backup collision. A backup collision is provided only when two particles move opposite to the direction dictated by the shearing flow. This is the reason why negative stress is a rare event and it is suppressed by the shearing field.

Inspection of Fig. 2 in the main part of the manuscript reveals that for a given packing fraction, ϕ, probability of the negative shear stress decreases upon increasing the shear rate. This can be justified by the fact that shearing favors the head-to-head collisions which result to positive shear stress. On the other hand, for a given shear rate, $\dot{\gamma}$, probability of the negative shear stress also decreases by increasing the packing fraction, ϕ. This can be justified by the fact that by increasing the packing fraction, one systematically increases coordination number of particles, z. At jamming density, ϕ_J, the mean coordination number is $z_c = 4$. For the fluid case $z < z_c$, and for the jammed phase $z \geq z_c$. Therefore, one can see that by increasing the packing fraction, number of contacts per particle will increase. Since the shearing suppresses backup collisions, increasing the coordination number of particles

will result to more head-to-head collisions. This results to a decrease of probability of negative events upon increasing the packing fraction.

Supplementary Note 5. BALANCE OF PROBABILITIES

Negative power $p < 0$ can occur for i) negative shear stress σ_{xy}^- and positive velocity gradient δv^+ or ii) negative velocity gradient δv^- and σ_{xy}^+. Therefore, probability for negative power, $P(p < 0)$, should be equal to the sum of the two joint probabilities of the aforementioned cases through

$$P(p < 0) = P(\sigma_{xy}^-, \delta v^+) + P(\delta v^-, \sigma_{xy}^+) \tag{16}$$

In Supplementary Fig. 3 we compile all our data to show the validity of this equation. Near jamming, where the negative events are suppressed, we obtain a large scatter due to large error bars.

Supplementary Note 6. TIME SCALE FOR THE FLUCTUATION RELATION

In the main part of the manuscript we conjecture a fluctuation relation which requires a time scale τ. In order to determine this time scale, we need to look again at the PDFs of the injected power (Fig. 1(**a**)-Inset in the main manuscript). As it was mentioned, for very small shear rates, the PDF becomes symmetric around $p = 0$. As a result, $P_{\dot{\gamma}\to 0}(p) \simeq P_{\dot{\gamma}\to 0}(-p)$ which gives zero slope for $\ln P(p)/P(-p)$ vs p. This is the lower limit of our fluctuation relation. On the other hand, for very large shear rates, the negative part of the PDFs diminish which results to approximately zero probability of negative power $P_{\dot{\gamma}\to\infty}(-p) \simeq 0$. This determines the upper limit of the shear rate above which the proposed fluctuation relation can not be applied. By a more careful look at our data, we decided the following range, $0.005 < \dot{\gamma} < 0.5$. In order to determine the relevant time scale in this range of shear rates, we look at the ratio of the elastic and the dissipative parts of the inter-particle forces. We observe that the former is larger than the latter by an order of magnitude. Therefore, in this range, the only relevant time scale comes from the elastic interactions, viz. $\tau = \tau_e$. The elastic time scale represents the typical time scale of a collision which is given by the

following relation

$$\tau_e = 0.5 \cdot \pi \cdot (\sqrt{\frac{m}{Y}} + \sqrt{\frac{M}{Y}}) \tag{17}$$

where m and M are masses of the small and large particles, respectively, and Y is the pre-factor of repulsive force in the linear Dashpot model. Each term in Supplementary Eq. 17 refers to half of the period of oscillation caused by the linear repulsive force.

Supplementary Note 7. CONSISTENCY OF CRITICAL EXPONENTS

A recent estimation of the critical exponents of jamming has been done by Vagberg et al. [3] in which the authors establish a scaling theory for the "average quantities" such as pressure p and shear stress σ in the system where the averages are taken over an ensemble of configurations in the steady state. Since p and σ are both components of a unified tensor, their scaling exponents y are assumed (and numerically shown) to be the same. The authors show that such average quantities follow a universal scaling form which, in the leading scaling form, is given by $\sigma = \dot{\gamma}^{y/z\nu} f(\delta\phi/\dot{\gamma}^{1/z\nu})$ with $f(x)$ being a universal scaling function and three critical exponents y, z and ν. Such scaling ansatz suggests that when the data for $\sigma/\dot{\gamma}^q$ with $q = y/z\nu$ is plotted versus $\delta\phi/\dot{\gamma}^{1/z\nu} = \delta\phi/\dot{\gamma}^{q/y}$ (or equivalently $\sigma/\delta\phi^y$ versus $\dot{\gamma}/\delta\phi^{y/q}$), all data at different values of ϕ and $\dot{\gamma}$ will collapse onto a single master curve. As stressed in the above paper, the exponent y is specific to the observable being measured, and indeed is so for q. Therefore, our exponents for T_e and T_g obtained in data collapse in Fig. 4 of our manuscript, need not necessarily be the same as those reported in the paper [3] for p and σ. On the other hand, the critical exponents should characterize the true algebraic divergence of the observables asymptotically close to the athermal jamming critical point, i.e., $\phi \to \phi_J$ and $\dot{\gamma} \to 0$ which basically needs simulations of very large system sizes. Since our fluctuation observation is specific to small system sizes we can not naturally approach to the true asymptotic region. In this sense, we can rather compare our findings with other studies in which the chosen parameters are close enough to ours.

Hatano [4–6] reported critical exponents of the granular temperature and shear stress in the range of shear rates similar to ours. According to these papers, our critical exponent $q = 1.5$ for T_g is in perfect agreement with that reported by Hatano for the kinetic temperature.

However, in order to establish a connection between the scaling behavior of our data

and those observed by Hatano (as an independent test for our data), we have measured ”average power flux” $P(\phi)$ as a function of the packing fraction ϕ for various shear rates $\dot{\gamma}$ and system size $L = 30$—see Main Supplementary Fig. 4. The vertical dashed line refers to the critical point $\phi_J = 0.84335(10)$ for the jamming transition reported in [3]. As shown in the Inset of Supplementary Fig. 4, the mean power flux at the jamming transition exhibits a scaling behavior with $\dot{\gamma}$, i.e., $P(\phi = \phi_J) \sim \dot{\gamma}^q$ with $q = 1.72(10)$. Our exponent q agrees with the critical exponent $y_\gamma = 0.63(2)$ reported in [6] for average shear stress (note that $P(\phi = \phi_J) \propto \sigma.\dot{\gamma} \sim \dot{\gamma}^{y_\gamma}.\dot{\gamma} \sim \dot{\gamma}^{1+y_\gamma}$). Since we adapt a more accurate critical point than that used by Hatano, our q exponent is slightly larger than the value reported by Hatano, but still in the same range within the numerical accuracy. Rescaled data for power flux with $\dot{\gamma}^q$, results in a plot in which all curves cross at the single critical jamming point—Main Supplementary Fig. 5. Now, in order to estimate the other critical exponent, we draw a horizontal line (the horizontal solid line in the Supplementary Fig. 5) which intersects all the curves and the vertical dashed line passing through the jamming point ϕ_J. We find that the distance between the intersection ϕ' for each curve and ϕ_J, shows a power-law behavior $|\phi' - \phi_J| \sim \dot{\gamma}^{q/y}$ with $q/y = 0.75(15)$—see Inset Supplementary Fig. 5. Using these two exponents, we find that all data collapse onto a single universal function when suitably rescaled—Supplementary Fig. 6.

Although the exponents q and y are specific to the corresponding observable which is measured, their ratio $q/y = 1/z\nu$ should be independent of the measured observable. In this sense our obtained exponent $q/y = 0.75(15)$ is larger than that obtained by Hatano as $q/y = 0.42(4)$. The reason is that here we demand a data collapse for a rather wide range of packing fractions far from the critical jamming point. In order to show that our exponents are in perfect agreement with those reported by Hatano, we focused on the critical region and generated more data around the critical point—Supplementary Fig. 7(**a**). We then estimated the exponent q/y around ϕ_J and find that $q/y = 0.49(10)$ in perfect agreement with that reported by Hatano withing the error bars—Supplementary Fig. 7(**b**).

Supplementary Note 8. VERIFICATION OF FR IN HIGHLY DISSIPATIVE REGIME

So far we have considered only quasi-elastic particles which correspond to granular materials. The value $t^\star = \gamma/Y = 3.15 \times 10^{-3}$ corresponds to the restitution coefficient $\epsilon \approx 0.98$

where particles are quasi-elastic. In order to account to non-Brownian suspensions, we also simulate highly dissipative particles. We have done simulations for $t^\star = \gamma/Y = 8.95 \times 10^{-2}$ where we only increased the value of the damping coefficient. This corresponds to a highly dissipative system with $\epsilon \approx 0.05$. In Supplementary Fig. 8, we depict our data for validation of our fluctuation relation for two different packing fractions $\phi = 0.7$ and 0.9. For both cases we are able to recover the fluctuation relation at the highly dissipative limit. One can see that similar to the quasi-elastic regime the slope (inverse of the effective temperature) decreases by increasing the shear rate $\dot{\gamma}$. Moreover, similar to the quasi-elastic limit, the slope change due to the shear rate for $\phi = 0.9$ (Supplementary Fig. 8(**b**) is weak.

Now, we test our scaling collapse of T_g and T_e for the case of highly dissipative systems. In Supplementary Fig. 9, we plot $T_g/|\delta\phi|^y$ and $T_e/|\delta\phi|^y$ vs $\dot{\gamma}/|\phi - \phi_c|^{y/q}$. Filled symbols refer to T_g and hollow symbols correspond to T_e. The color code show different shear rates. We adopt here exactly the same exponents and critical points as we used for the quasi-elastic systems. One can see that we obtain a similar scaling collapse. This enables us to conclude that the critical exponents describing scaling behavior of T_e and T_g are universal in a sense that they are independent of the damping coefficient and system size.

SUPPLEMENTARY REFERENCES

[1] Schwager, T. & Pöschel, T. Coefficient of restitution and linear dashpot model revisited. *Granular Matter* **9**, 465 (2007).

[2] O'Hern, C., Silbert, L., Liu, A. & Nagel, S. Jamming at zero temperature and zero applied stress: The epitome of disorder. *Phys. Rev. E* **68**, 011306 (2003).

[3] Vagberg, D., Olsson, P. & Teitel, S. Critical scaling of Bagnold rheology at the jamming transition of frictionless two-dimensional disks. *Phys. Rev. E* **93**, 052902 (2016).

[4] Hatano, T., Otsuki, M. & i. Sasa, S. Criticality and scaling relations in a sheared granular material. *J. Phys. Soc. Jpn.* **76**, 023001 (2007).

[5] Hatano, T. Scaling properties of granular rheology near the jamming transition. *J. Phys. Soc. Jpn.* **77**, 12 (2008).

[6] Hatano, T. Critical scaling of granular rheology. *Progr. Theor. Exp. Phys.* **184**, 143 (2010).
