# Supplementary material for: Characterizing rare fluctuations in soft particulate flows
Source: Nat Commun. 2017 Apr 10;8:11. doi: 10.1038/s41467-017-00022-8 (PMC5431914; doi:10.1038/s41467-017-00022-8)
Supplement: Supplementary file 2 — Reviewer reports and authors' response from the peer review of this Article at Nature Communications [file 41467_2017_22_MOESM2_ESM.pdf]

Reviewers' comments:

Reviewer #1 (Remarks to the Author):

Dear editor,

I have carefully reviewed manuscript NCOMMS-16-26133-T "Characterizing rare fluctuations in soft particulate flows" by S. H. E. Rahbari, A. A. Saberi, H. Park, and J. Vollmer. This manuscript describes computer simulations of sheared frictionless disks to understand the nature of power fluctuations above and below the jamming packing fraction. The authors show an interesting minimum in the negative power flucutations near the jamming transition, decompose the negative power fluctuations in terms of contributions from negative stress or negative velocity differences, identify an effective temperature T_e from a power fluctuation relation, and achieve a scaling collapse of the effective temperature T_e over a wide range of shear rate and packing fraction.

This manuscript is well written and shows very interesting new results on negative power fluctuations near the jamming transition in two-dimensional frictionless disks, which can model vibrated granular systems, foams, and other particulate media. This work will motivate future studies of power fluctuations and effective temperatures in both simulations and experiments of particulate media near the jamming transition.

I strongly recommend publication of the manuscript after the authors address the following comments:

1. The authors show a nice scaling collapse of the effective temperature T_e with shear rate and packing fraction with scaling exponents y and q. The authors should compare their exponents to those (q and 1/(z \nu)) obtained for the collapse of the shear stress with shear rate and packing fraction by Vagberg, et al. (Phys. Rev. E 93 (2016) 052902). Can the two sets of exponents be made consistent with each other?

2. The authors mention other measurements of effective temperatures in systems of 2D and 3D frictionless disks that used linear response relations rather fluctuation relations. The authors state on the bottom of page 3 that effective temperatures from fluctuation relations are valid for packing fractions both far and near the jamming packing fraction, whereas those based on linear response are only valid near the jamming point. This statement has not been sufficiently justified by the current studies. In particular, I don't see why the authors state that linear response relations are more valid near jamming onset.

3. Related to point 2, it would be great if the authors could measure one of the previously studied effective temperatures (defined from linear response) and compare the scaling collapse to that of T_e. This measurement would address the extent to which the assumption of linear response affects the scaling behavior of power or stress fluctuations near phi_J.

Reviewer #2 (Remarks to the Author):

Rahbari et. al. Characterizing rare fluctuations in soft particulate flows

The authors study the distribution of energy dissipation in a (driven, dissipate) model system near jamming. Their main statistical tools are i) the fraction of the time the system spends anti-dissipating energy and ii) the ratio of the fraction of the time, P(p) the system is dissipating a given amount of energy, p, to the fraction P(-p) of the time it is anti-dissipating the same amount. They define an effective temperature, T_e, using the slope of the Ln[P(p)/P(-p)]. They study T_e as a function shearing rate and volume fraction showing that the jamming transition serves as a critical point allowing for a scaling collapse of the T_e data.

I think these are tremendously important and novel ideas that could have a large impact across soft-matter and statistical physics. I am very enthusiastic about the general approach, however there are many technical issues which need to be addressed. These technical issues could potentially prevent publication.

+ Heussinger, Chaudhuri, and Barrat have looked at velocity flcutations above and below jamming in quasi-static simulations. They find pronounced system size effects in the velocity fluctuations above phi_J. I expect the authors would find corresponding size effects in their T_e above phi_J (at least at sufficiently small rates). Have they checked this? (Please cite Heussinger and Barrat Phys. Rev. Lett. 102, 218303 and Heussinger, Chaudhuri, and Barrat Soft Matter, 2010, 6, 3050–3058) Can the authors convince me that their definition of T_e is not size depedent in the jammed branch above phi_J? Also, it seems like the scaled flow curve is non-monotonic at the lowest rate: does T_e really increase at decreasing rate???. I think this just means that the collapse must be failing in that regime, but it’s hard to tell, since they only show data for a single rate in that regime of deltaPhi. It also looks like the collapse is pretty bad in general for rate=0.02. It would be good to check to see if they can get a reasonable collapse with runs at fixed THIS IS MY MOST SERIOUS TECHNICAL CONCERN AND IT MUST ABSOLUTELY BE ADDRESSED BEFORE PUBLICATION.

+ The authors should cite Maloney (Maloney 2015 EPL 111 28001) who performs a very closely related study on Durian’s bubble model close to, but just above, phi_J. The main finding there is that P(p) has a power law form at sufficiently low rates. The power law appears at any fixed phi above phi_J as sharing rate->0 and disappears at any fixed rate as phi->phi_c. These power-law distributions would cause strongly non-exponential behavior in the ratio plot Fig3b and would make it difficult to define T_e from those plots. In fact, it seems like Fig3b is not quite so exponential as the authors would like the reader to believe. They should explain this.

+ They never define granular temperature. This is not a triviality, since in the non-inertial limit where their t_* parameter goes to infinity (and their model becomes Durian’s bubble model), there is no inertial contribution to the stress or energy, so it is not possible to define granular temperature.

+ The authors are never clear about how they measure the local stress and velocity fluctuations (e.g. used to generate Fig2). Does the stress they measure include potential (from the springs), viscous (from the dashpot), and inertial contributions? Is this a particle-by-particle measurement or is there some coarse-graining, and, if so, how is it done?

+ This (coarse-graining) will crucially affect the statistics. The histogram they show in Fig1 inset will get narrower if they average the stress and velocity fluctuations over larger regions. The distributions will also get narrower with increased sampling time rather than looking at instantaneous dissipation.

+ Really, my previous two bullet points make me wonder why the authors even show Fig2 based on the LOCAL fluctations. As far as I read it, this does not enter into any of the subsequent discussion on scaling behavior of T_e near the phi->phi_J shearRate->0 critical point.

+ They choose t_*= \gamma/Y=3.15 × 10−3. That is a time measured in units of the inertial timescale. The damping is so weak that this is really a simulation of a "granular material" rather than a "soft-particle suspension". Most of the previous work on effective temperatures (ref 24 and references therein) used Durian's bubble model which is effectively the authors' model locked at t_*=infinity. Would they expect the same results in the limit of very large t_* corresponding to an overdamped non-Brownian suspension rather than an inertial granular flow?

+ They almost completely ignore the rich literature on fluctuation theorems in non-driven Hamiltonian or Langevin systems by Gallavotti and Cohen, Jarzynski, Crooks, and others. In this literature the energy dissipation (or entropy production) along trajectories depends crucially on the sampling time. Of course, the 2nd law of thermodynamics must be obeyed (in Hamiltonian systems) for large numbers of particles and at long sampling times. In the authors' terminology, "obey 2nd law" means p>0, or "entropy is always created and never destroyed". But even large systems can spontaneously destroy entropy (p<0) if the statistics dissipation/anti-dissipation (entropy creation/destruction) are taken on short engouh time intervals. They say nothing about this time interval in their study and look only at instantaneous values of p. It would be interesting to at least mention how their work connects with the more general Gallavotti-Cohen fluctuation theorems where the duration of trajectories (in addition to system size) is key.

0.1 Response to referee 3

Upon our communications, we learned that the third referee has decided to withdraw from the review process as his/her concerns are covered by the other two reviewers. May we add request to convey our thanks to the referee for taking his/her time to review our manuscript.

0.2 Response to referee 2

On behalf of all my co-authors, I would like to express my gratitude to your positive attitude towards our manuscript and your credible comments. We especially thank for your encouraging remarks:

"*I think these are tremendously important and novel ideas that could have a large impact across soft-matter and statistical physics. I am very enthusiastic about the general approach...*"

We found your comments very insightful that helped us to further develop our manuscript. We went through all your questions and below we provide a detailed response to your comments. We have acknowledged your insights in the paper.

0.2.1 Comment 1

Referee's comment:

"Heussinger, Chaudhuri, and Barrat have looked at velocity fluctuations above and below jamming in quasi-static simulations. They find pronounced system size effects in the velocity fluctuations above ϕ_J. I expect the authors would find corresponding size effects in their T_e above ϕ_J (at least at sufficiently small rates). Have they checked this? (Please cite Heussinger and Barrat Phys. Rev. Lett. 102, 218303 and Heussinger, Chaudhuri, and Barrat Soft Matter, 2010, 6, 30503058) Can the authors convince me that their definition of T_e is not size dependent in the jammed branch above ϕ_J? Also, it seems like the scaled flow curve is non-monotonic at the lowest rate: does T_e really increase at decreasing rate???. I think this just means that the collapse must be failing in that regime, but its hard to tell, since they only show data for a single rate in that regime of $\delta\phi$. It also looks like the collapse is pretty bad in general for $\dot{\gamma} = 0.02$. It would be good to check to see if they can get a reasonable collapse with runs at fixed THIS IS MY MOST SERIOUS TECHNICAL CONCERN AND IT MUST ABSOLUTELY BE ADDRESSED BEFORE PUBLICATION."

Response, first part: Thanks for suggesting these interesting and related papers to our work. We have cited these papers in the manuscript. In order to investigate the finite-size effects, we have done new simulations for system sizes $L = 10$, 20, 50, and 80 for packing fractions below and above jamming ϕ_J and different range of shear rates $\dot{\gamma}$. In Fig. 1 we compile our data for the effective temperature as a function of the packing fraction and shear rate for different system sizes. Different system sizes are given by different symbols which are depicted in the legend. The color code corresponds to $\dot{\gamma} = 0.02$ (purple), 0.04 (green), 0.06 (blue), 0.08 (golden), and 0.10 (yellow).

We revisited Fig. 4 in the main part of the manuscript by adding different system sizes to the scaling collapse of the effective and granular temperature.

We added this paragraph to the manuscript:

Heussinger *et al.* [Heussinger, Chaudhuri, and Barrat Soft Matter, 2010, 6, 30503058] have studied fluctuations of some observables in the flow of an assembly of frictionless, soft discs at zero temperature, in the vicinity of and slightly above ϕ_J. They have found that the contact-number fluctuations and relative fluctuations of the shear stress diverge upon approaching ϕ_J from above. They also report on strong finite-size effects when using ϕ as control parameter. However, the effective temperature T_e in our study, is the product of thermodynamically forbidden fluctuations (negative stress below ϕ_J and negative velocity gradient above ϕ_J) which are specific to small-size systems and vanish in the vicinity of ϕ_J. Our observation indicates that the scaling behavior of T_e is not significantly altered by the finite-size effects below and above ϕ_J for $L > 10$— see Fig. 4. We find that to a very good extent, T_e is independent of the system size. We only see small deviations for $L = 10$. These deviations vanish as $\dot{\gamma} \to 0$.

Response, second part: Flow curves are indeed monotonic for all packing fractions. In order to demonstrate it, we plot the un-scaled effective temperature T_e, which is used in the previous figure, as function of the shear rate for all packing fractions (Fig. 2). One can see that for all packing fractions the effective temperature is a monotonically increasing function of the shear rate. The fact that $\dot{\gamma} = 0.02$ (purple curve) in Fig. 1 seems to be non-monotonic in the jammed state is that both nominator and denominator in $T_e/|\delta\phi|^y$ vary from one point to

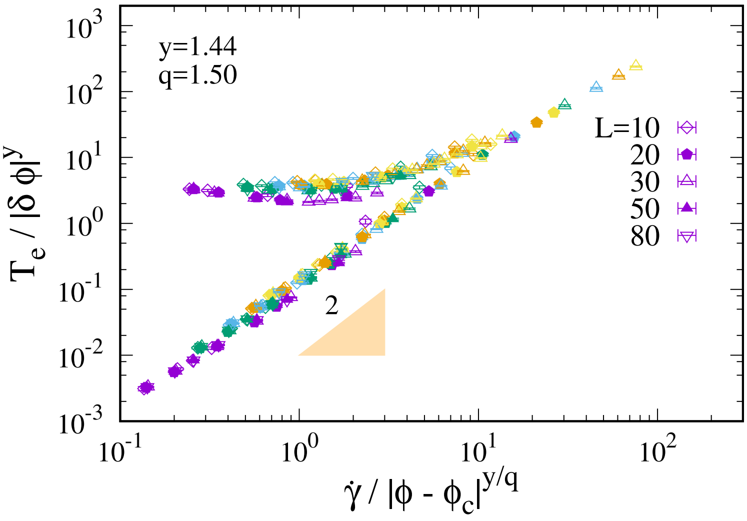

Figure 1: **Scaling collapse of effective temperature T_e for different system sizes.** Scaling of the effective temperature T_e as function of the shear rate $\dot{\gamma}$ and the packing fraction ϕ for different system sizes $L = 10$, 20, 30, 50, and 80. Each symbol corresponds to a different system size. The color code refers to $\dot{\gamma} = 0.02$ (purple), 0.04 (green), 0.06 (blue), 0.08 (golden), and 0.10 (yellow). One can see that all different system sizes have the same effective temperature below and above jamming ϕ_J. We only see small deviations for $L = 10$ which vanishes as $\dot{\gamma} \to 0$. The scaling collapse is achieved by adopting the same exponents and critical density for all different system sizes. Critical exponents are $q = 1.5$ and $y = 1.44$ and the critical density is $\phi_J = 0.84$.

another. This makes the latter quantity non-monotonic.

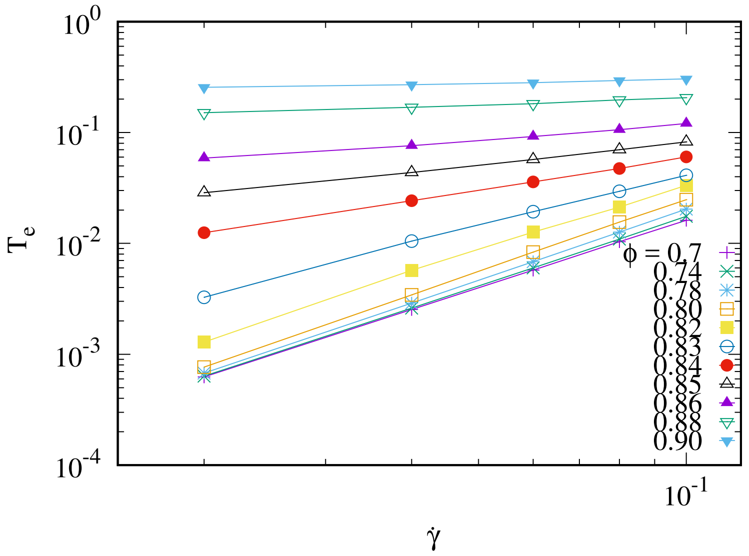

Figure 2: **Un-scaled effective temperature $\mathbf{T_e}$.** The effective temperature vs shear rate for different packing fractions. The system size is $L = 30$. The effective temperature T_e is a monotonically increasing function of the shear rate.

0.2.2 Comment 2

Referee's comment:

"The authors should cite Maloney (Maloney 2015 EPL 111 28001) who performs a very closely related study on Durians bubble model close to, but just above ϕ_J. The main finding there is that $\mathcal{P}(p)$ has a power law form at sufficiently low rates. The power law appears at any fixed phi above ϕ_J as $\dot{\gamma} \to 0$ and disappears at any fixed rate as $\phi \to \phi_c$. These power-law distributions would cause strongly non-exponential behavior in the ratio plot Fig3b and would make it difficult to define T_e from those plots. In fact, it seems like Fig3b is not quite so exponential as the authors would like the reader to believe. They should explain this."

Response: Thanks a lot for introducing this interesting reference. We have cited this paper in the manuscript. In this paper distribution of *dissipation power* $\mathcal{P}(\Gamma)$ in the Durian's bubble model is investigated. It is indeed a very interesting point to investigate equality of higher moments of distributions of *dissipation power* $\mathcal{P}(\Gamma)$ and *injection power* $\mathcal{P}(p)$ in soft particulate flows. This paper together with our paper provide a framework for such analysis. To the best of our knowledge, this point has been overlooked in the literature.

We have added this paragraph to the paper:

In a recent study, Maloney [4] investigated distribution of dissipation power $\mathcal{P}(\Gamma)$ in Durian's bubble model. Whereas we find that $\mathcal{P}(p)$ has always exponential tails, it is shown that above jamming density $\mathcal{P}(\Gamma)$ becomes power law for small shear rates. This implies that distributions of injection and dissipation powers might not not be equivalent. Stationarity condition implies that first moments of these distributions must be equal. But as one can see, higher moments of these distributions, which refer to the characteristic of the tails, might be different. This suggests a new avenue of research for investigation of steady state properties of non-equilibrium states. In this context, Maloney's work [4] together with our approach to calculate $\mathcal{P}(p)$ in shear flows provide a solid framework for investigation of similarities and differences between distributions of injection and dissipation powers.

In Fig. 3 distribution of power $\mathcal{P}(p)$ is plotted for different packing fractions above jamming for a fixed shear rate $\dot{\gamma} = 0.01$. One can see that all distributions display exponential tails. Moreover, we only observe exponential tails for all packing fractions above the jamming. In the case of fat tails, one can also derive an effective temperature near $p \to 0$ where the ratio of probabilities are normally exponential. However, interpretation of such slope can be disputed. For smaller shear rates, $\dot{\gamma} < 0.01$, we observe similar distributions.

In general, there are many reasons that give rise to leveling off in the plot of $\ln[P(p)/P(-p)]$ vs p for large p. Below, we will explain two relevant reasons which result to a bend for large p.

I. Neglecting the system entropy change: In the usual fluctuation theorems, the total entropy change ΔS_{tot} satisfies:

$$\ln[P(\Delta S_{tot})/P(-\Delta S_{tot})] = \Delta S_{tot} \tag{1}$$

where

$$\Delta S_{tot} = \Delta S_{sys} + \Delta S_{res} \tag{2}$$

in which ΔS_{sys} and $\Delta S_{res} = Q/T$ are changes of the entropy of the system and reservoir, respectively. If change of the entropy of system is neglected, still ΔS_{res} satisfies a fluctuation

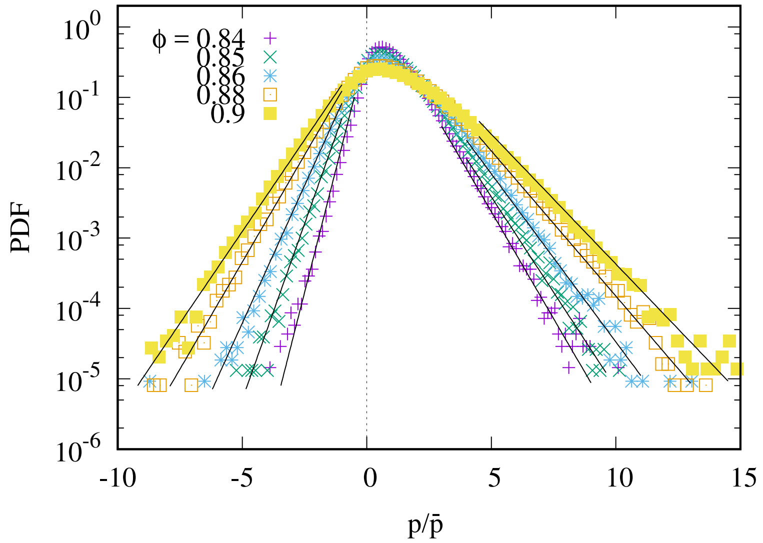

Figure 3: **PDF of injected power p.** Probability distribution of the injected power for the jammed state at fixed shear rate $\dot{\gamma} = 0.01$. Different colors refer to different packing fractions $\phi = 0.84$ (purple), 0.85 (green), 0.86 (blue), 0.88 (golden), and 0.90 (yellow). For all cases we find exponential tails. Solid lines are exponential fits as guides for eye. The system size is $L = 30$.

relation of the form:

$$\ln[P(\Delta S_{res})/P(-\Delta S_{res})] = A(Q)Q/T \tag{3}$$

where $A(Q)$ is a function of the dissipated heat Q, and T is the reservoir temperature. For small Q, the prefactor $A(Q)$ is normally equal to unity. However, for larger values of the dissipated heat Q, the prefactor results to a saturation at higher Q.

Here, in our study the injected power is the entropy production rate of the system and we *inevitably ignore* the entropy production change in the internal degrees of freedom of particles. This naturally will give rise to a small saturation for higher values of p.

II. Poor statistics at the tails: Probability of negative power at p is proportional to the number of encounters for that particular bin $[-p, -p - \delta p]$, *i.e.*, $P(p) \propto N^-/N$. It is often the case for numerical and experimental data that statistics becomes poor at the tails ($N^- \ll N$). This results to a saturation of $\ln[P(p)/P(-p)]$ vs p for large p. In order to make a clean estimation of slopes, we systematically neglect points where $N^- < 60$. This becomes more pronounced near ϕ_J where negative events are suppressed.

0.2.3 Comment 3

Referee's comment:

"They never define granular temperature. This is not a triviality, since in the non-inertial limit where their $t^\star$ parameter goes to infinity (and their model becomes Durians bubble model), there is no inertial contribution to the stress or energy, so it is not possible to define granular temperature.

Response: By definition we have always the inertial term in the microscopic equations of motion. Indeed, it is very interesting to repeat our procedure for Durian's bubble model where particles have no inertia. We have added this part in the Supplementary Information to define granular temperature:

Granular temperature T_g is defined as the *random* kinetic energy of particles. Here, the randomness means that the drift velocity is subtracted from velocity of each particle, so that the temperature can be defined as:

$$T_g = \frac{1}{N}\sum_{i=1}^{N}\frac{1}{2}m_i\left(\langle(v_{i,y} - v_y(x))^2\rangle\right) \tag{4}$$

where the sum over i runs over N number of particles.

0.2.4 Comment 4

Referee's comment:

" The authors are never clear about how they measure the local stress and velocity fluctuations (e.g. used to generate Fig2). Does the stress they measure include potential (from the springs), viscous (from the dashpot), and inertial contributions? Is this a particle-by-particle measurement or is there some coarse-graining, and, if so, how is it done?"

Response: We have added this part in the Supplementary Information to define how we measure stress tensor and average it over bins (we only consider the spring (potential) force in the calculation of the stress tensor):

The system is divided into narrow rectangular bins of area $2 \times L$ along the flow direction and thus $L/2$ stripes in each simulation box. The aim of this is to measure relevant physical quantities locally as function of the spatial position of the bin. Due to the Lees-Edwards boundary condition imposed along the x-direction, the average velocity profile depends only on x, and thus for a given bin at each position x, all local averages for the quantities of interest are taken over the particles in the y direction. During each simulation, we take 350 snapshots of all local physical quantities inside the bins. Quantities of interest are the shear stress σ_{xy}, the local drift velocity $v_y(x)$, and the granular temperature T_g (definition of these quantities are given in the next section).

We run 100 different realizations for each set of parameters. In the main paper, the averaged quantities such as shear stress σ_{xy}, and the injected-power p, are thus taken over 100×350 snapshots; each of which consists of $L/2$ entries for different bins. For example, for $L = 50$, we get 875×10^3 data points. To probe the aforementioned range of parameters, we performed altogether 20000 individual simulations; and on average one day of CPU-time for each simulation.

Let us first define the shear stress σ_{xy} which is often measured and used in our calculations. At the spatial position x, the local shear stress $\sigma_{xy}(x)$ is calculated according to

$$\sigma_{xy}(x) = \frac{1}{A} \sum_i \frac{1}{2} m_i v_{i,x} \times (v_{i,y} - v_y(x)) + \frac{1}{A} \sum_{i<j} r_{ij,x} \times F_{ij,y}, \tag{5}$$

where $A = 2 \cdot L$ is the area of of the rectangular bin at position x, $v_{i,x}$ and $v_{i,y}$ are the x and y components of the velocity of the ith particle, respectively, $v_y(x)$ is the local drift velocity at x, $r_{ij,x}$ is the x component of the vector which connects the center of particle i to that of particle j, and $F_{ij,y}$ is the y component of the vector force exerted by particle i to j. We only consider the spring (potential) force in the calculation of the stress tensor. The first sum in the right hand side of Eq. (5) runs through all particles at spatial position x, and the second sum runs over all neighboring particles that interact with particle i. If the neighboring particle belongs to a neighboring bin, we only consider half of the second term for each bin. With this local shear stress, it is straightforward to calculate the global shear stress Σ_{xy} in the system:

$$\Sigma_{xy} = \frac{1}{L} \int_0^L \sigma_{xy}(x) dx. \tag{6}$$

0.2.5 Comment 5

Referee's comment:

"This (coarse-graining) will crucially affect the statistics. The histogram they show in Fig.1 inset will get narrower if they average the stress and velocity fluctuations over larger regions. The distributions will also get narrower with increased sampling time rather than looking at instantaneous dissipation."

Response: The referee is perfectly right. The second law of thermodynamics, usually known as some inequalities, always remains true only *in average.* As we turn our attention to smaller systems with a few degrees of freedom, statistical fluctuations become more prominent and the second law becomes increasingly blurred. This means that such inequalities are not mandated by the underlying microscopic dynamics: There certainly exist microscopically viable N-particle trajectories for which the inequalities are violated. However, the probability to observe such trajectories becomes fantastically small for large system sizes [C. Jarzynski, Annu. Rev. Condens. Matter Phys. 2011.2:329-351].

Therefore, when we take averages over a large sample size or along a long time interval, the *thermodynamically forbidden* fluctuations are washed out and the negative part in the histogram shown in Fig. 1 gets narrower and finally disappears for large enough system sizes. This is why we focus on characterizing rare fluctuations in small systems and avoid making any averaging which cause the loss of information. In fact we chose a situation in which our approach would remain as general as possible: We look at local instantaneous (NO time averaged or coarse-grained) quantities at smallest possible length scale, i.e., at a transverse length scale (along x-axis) where effectively one contact point with possible collisions can be observed. That's why we set the width of the bins equal to 2 mean particle diameter. Moreover, the only averages are taken along the vertical (y-axis) direction within which the velocity profile $v_y(x)$ is constant in average. Thus we believe that our approach is based on instantaneous microscopic observation of rare events which is general and without any controlling parameter. One will absolutely loose information about the fluctuations (as the referee correctly pointed out) as long as working with coarse-grained quantities.

Our argument is best supported by Fig. 1 where data of effective temperatures from different system sizes sit on top of each other on the scaling collapse with the same critical density and critical exponents.

0.2.6 Comment 6

Referee's comment:

"Really, my previous two bullet points make me wonder why the authors even show Fig.2 based on the LOCAL fluctuations. As far as I read it, this does not enter into any of the subsequent discussion on scaling behavior of T_e near the $\phi \to \phi_J$ and $\dot{\gamma} \to$ critical point."

Response: The effective temperature T_e is defined according to a fluctuation relation for the local instantaneous fluctuations of the negative power flux below and above the critical jamming point as a function of ϕ . Although Fig. 2 seems not computationally to contribute in the scaling behavior of T_e, but it unravels from the inherent nature of the mutually exclusive contributions in the negative power fluctuations: Below ϕ_J , negative shear stress dominates, while above ϕ_J, the reversal of the velocity gradient has a dominant contribution. It provides an intuitive understanding of the jamming transition during which the responsible observable for the negative power changes. In fact, it shows that below ϕ_J the effective temperature is defined in terms of the statistics of the negative shear stress while above the jamming transition, T_e is defined by the statistics of the negative velocity gradient.

0.2.7 Comment 7

Referee's comment:

"They choose $t^\star = \gamma/Y = 3.15 \times 10^{-3}$. That is a time measured in units of the inertial timescale. The damping is so weak that this is really a simulation of a granular material rather than a soft-particle suspension. Most of the previous work on effective temperatures (ref 24 and references therein) used Durians bubble model which is effectively the authors model locked at $t^\star = \infty$. Would they expect the same results in the limit of very large $t^\star$ corresponding to an overdamped non-Brownian suspension rather than an inertial granular flow?"

Response: Thanks for suggesting us to test our proposed fluctuation relation for a highly damped system. Indeed, $t^\star = \gamma/Y = 3.15 \times 10^{-3}$ corresponds to the restitution coefficient $\epsilon \approx 0.98$ where particles are almost quasi-elastic. We have done new simulations for $t^\star = \gamma/Y = 8.95 \times 10^{-2}$ where we only increased the value of the damping coefficient. This corresponds to a highly dissipative system with $\epsilon \approx 0.05$. In Fig. 4, we depict our data for verification of our fluctuation relation for two different packing fractions $\phi = 0.7$ and 0.9. For both cases we are able to recover the fluctuation relation at the highly dissipative limit. One can see that similar to the quasi-elastic regime the slope (inverse of the effective temperature) decreases by increasing the shear rate $\dot\gamma$. Moreover, similar to the quasi-elastic limit, the slope change due to the shear rate for $\phi = 0.9$ (Fig. 4-b) is weak.

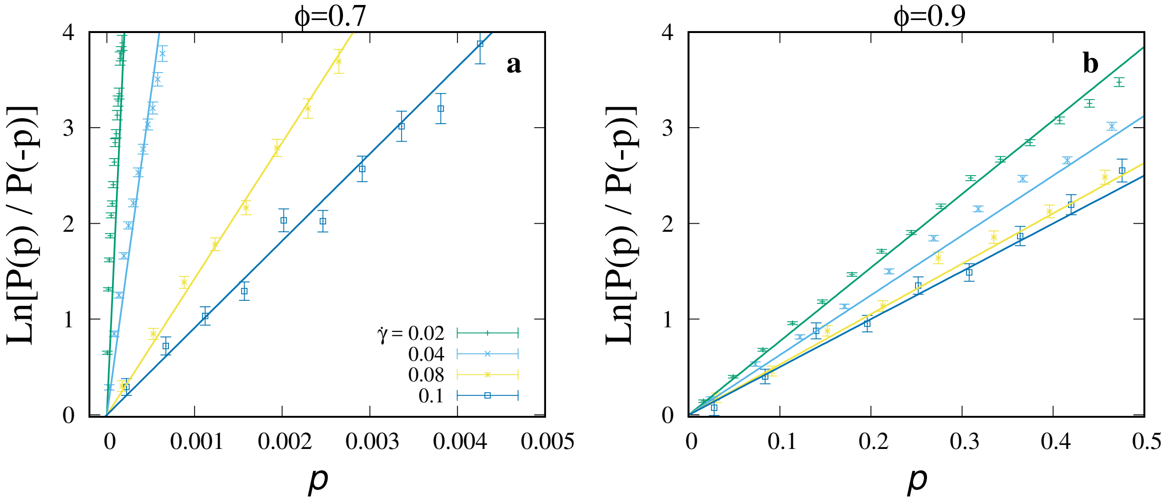

Figure 4: **Verification of FT for highly dissipative system.** Plot of $Ln[P(p)/P(-p)]$ vs p for two packing fractions $\phi = 0.7$ (left) and $\phi = 0.9$ (right). The damping coefficient is $\gamma = 8.95$ and the spring prefactor is $Y = 100$. This corresponds to a highly dissipative system. The solid lines are linear fits of slope $\beta_e = \tau/T_e$ to the data for different shear rates where T_e is the effective temperature and τ is the refined time scale. The slope decreases by increasing the shear rate $\dot\gamma$, implying that the effective temperature T_e increases by $\dot\gamma$. The slope has a weak dependence on $\dot\gamma$ in the jammed state.

Now, we test our scaling collapse of T_g and T_e for the case of highly dissipative systems. In Fig. 5, we plot $T_g/|\delta\phi|^y$ and $T_e/|\delta\phi|^y$ vs $\dot\gamma/|\phi-\phi_c|^{y/q}$. Filled symbols refer to T_g and hollow symbols correspond to T_e. The color code show different shear rates. We adopt here exactly the same exponents and critical points as we used for the quasi-elastic systems in Fig. 1. One can see that we obtain a similar scaling collapse. This enables us to conclude that the critical exponents describing scaling behavior of T_e and T_g are universal in the sense that they are

independent from the damping coefficient and system size. To the best of our knowledge, this is the first numerical proof for such universality.

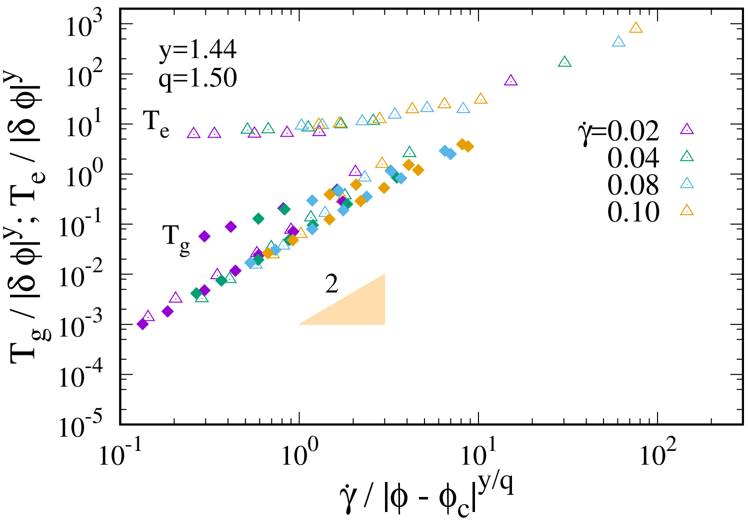

Figure 5: **Scaling collapse of T_e and T_g for highly dissipative system.** $T_g/|\delta\phi|^y$ and $T_e/|\delta\phi|^y$ vs $\dot{\gamma}/|\phi - \phi_c|^{y/q}$ are plotted. Filled symbols refer to T_g and hollow symbols correspond to T_e. The color code shows different shear rates. Here, we adopt exactly the same exponents and critical densities as we used for quasi-elastic systems in Fig. 1. This demonstrates the robustness of universality against changes in the dissipation in the system. The damping coefficient is $\gamma = 8.95$ and the spring constant is $Y = 100$.

0.2.8 Comment 8

Referee's comment:

"They almost completely ignore the rich literature on fluctuation theorems in non-driven Hamiltonian or Langevin systems by Gallavotti and Cohen, Jarzynski, Crooks, and others. In this literature the energy dissipation (or entropy production) along trajectories depends crucially on the sampling time. Of course, the 2nd law of thermodynamics must be obeyed (in Hamiltonian systems) for large numbers of particles and at long sampling times. In the authors terminology, obey 2nd law means $p > 0$, or entropy is always created and never destroyed. But even large systems can spontaneously destroy entropy ($p < 0$) if the statistics dissipation/anti-dissipation (entropy creation/destruction) are taken on short enough time intervals. They say nothing about this time interval in their study and look only at instantaneous values of p. It would be interesting to at least mention how their work connects with the more general Gallavotti-Cohen fluctuation theorems where the duration of trajectories (in addition to system size) is key."

Response: Many thanks for bringing our attention to this crucial point. We added a paragraph to the manuscript where we discuss similarities and differences between our proposed fluctuation relations with the existing fluctuation theorems.

The paragraph in the manuscript is as follows:

Various types of fluctuation theorems have been extensively studied over the last two decades [Udo Seifert, Rep. Prog. Phys. 75 (2012) 126001; C. Jarzynski, Annu. Rev. Condens. Matter Phys. 2011.2:329-351. G. E. Crooks, Phys. Rev. E 60, 2721 (1999); C. Jarzynski, Phys. Rev. Lett. 78, (1997).]. Motivated by molecular dynamics simulations, Evans *et al.* [10] proposed an empirical fluctuation relation for entropy production rate in a 2-dimensional sheared Lennard-Jones fluid. Later, this empirical relation was rigorously proved by Gallavotti and Cohen [5, 6]. This is now known as steady-state fluctuation theorem (SSFT). In a steady-state fluctuation theorem, the entropy production rate is time averaged over a single, randomly sampled interval of duration τ. In contrast, the transient fluctuation theorem (TFT) of Evans and Searles [13] applies to a system that evolves from an initial equilibrium state to a nonequilibrium steady state. TFTs are different from SSFTs from a practical point of view. Whereas TFRs rely on ensemble averaging all starting from the same initial macro-state, SSFRs may be verified from steady-state evolution of a system over a sufficiently long time [9]. As we have already stressed out, properties of soft particulate flows are predominated by the rare fluctuations which result to intermittent behavior of these flows. It can be seen that fluctuation relations of type SSFTs are not suitable for soft particulate flows. The reason is that as a consequence of averaging process during the sampling time, the rare fluctuations can be washed out. Therefore, we use an instantaneous, time-independent fluctuation relation to characterize strength of rare fluctuations. Whether our postulated fluctuation relation would enjoy a rigorous treatment, will remain a theoretical challenge.

0.3 Response to referee 1

On behalf of all my co-authors, let me express my gratitude to your positive attitude towards our manuscript and your valuable comments. We especially thank for your generous remarks:

"*This manuscript is well written and shows very interesting new results on negative power fluctuations near the jamming transition in two-dimensional frictionless disks, which can model vibrated granular systems, foams, and other particulate media. This work will motivate future studies of power fluctuations and effective temperatures in both simulations and experiments of particulate media near the jamming transition. I strongly recommend publication of the manuscript after the authors address the following comments ...*

We found your comments very insightful that helped us to further develop our manuscript. We went through all your questions and below we provide a detailed response to your comments. We have acknowledged your inputs in the paper.

0.3.1 Comment 1

Referee's comment:

"The authors show a nice scaling collapse of the effective temperature T_e with shear rate and packing fraction with scaling exponents y and q. The authors should compare their exponents to those (q and $1/(z\nu)$) obtained for the collapse of the shear stress with shear rate and packing fraction by Vagberg, et al. (Phys. Rev. E 93 (2016) 052902). Can the two sets of exponents be made consistent with each other?"

Response: We would like to thank the referee for his/her very relevant comment. In the work [Phys. Rev. E 93 (2016) 052902], the authors establish a scaling theory for the "*average quantities*" such as pressure p and shear stress σ in the system where the averages are taken over an ensemble of configurations in the steady state. Since p and σ are both components of a unified tensor, their scaling exponents y are assumed (and numerically shown) to be the same. The authors show that such average quantities follow a universal scaling form which, in the leading scaling form, is given by $\sigma = \dot{\gamma}^{y/z\nu} f(\delta\phi/\dot{\gamma}^{1/z\nu})$ with $f(x)$ being a universal scaling function and three critical exponents y, z and ν. Such scaling ansatz suggests that when the data for $\sigma/\dot{\gamma}^q$ with $q = y/z\nu$ is plotted versus $\delta\phi/\dot{\gamma}^{1/z\nu} = \delta\phi/\dot{\gamma}^{q/y}$ (or equivalently $\sigma/\delta\phi^y$ versus $\dot{\gamma}/\delta\phi^{y/q}$), all data at different values of ϕ and $\dot{\gamma}$ will collapse onto a single master curve. As stressed in the above paper, the exponent y is specific to the observable being measured, and indeed is so for q. Therefore, our exponents for T_e and T_g obtained in data collapse in FIG. 4 of our manuscript, need not necessarily be the same as those reported in the paper [Phys. Rev. E 93 (2016) 052902] for p and σ. On the other hand, the critical exponents should characterize the true algebraic divergence of the observables asymptotically close to the athermal jamming critical point, i.e., $\phi \to \phi_J$ and $\dot{\gamma} \to 0$ which basically needs simulations of very large system sizes. Since our fluctuation observation is specific to small system sizes we can not naturally approach to the true asymptotic region. In this sense, we can rather compare our findings with other studies in which the chosen parameters are close enough to ours.

Hatano [1, 2, 3] reported critical exponents of the granular temperature and shear stress in the range of shear rates similar to ours. According to this paper, our critical exponent $q = 1.5$ for T_g is in perfect agreement with that reported by Hatano for the kinetic temperature.

However, in order to establish a connection between the scaling behavior of our data and those observed in the paper mentioned by the referee and the work of Hatano (as an independent test for our data), we have measured "*average* power flux" $P(\phi)$ as a function of the packing fraction ϕ for various shear rates $\dot{\gamma}$ and system size $L = 30$—see Main Fig. 6. The vertical dashed line refers to the critical point $\phi_J = 0.84335(10)$ for the jamming transition reported in the mentioned paper by Vagberg, et al. As shown in the Inset of Fig. 6, the mean power flux at the jamming transition exhibits a scaling behavior with $\dot{\gamma}$, i.e., $P(\phi = \phi_J) \sim \dot{\gamma}^q$ with $q = 1.72(10)$. Our exponent q agrees with the critical exponent $y_\gamma = 0.63(2)$ reported in [T. Hatano, Progr. Theor. Exp. Phys., 184 143 (2010)] for average shear stress (note that $P(\phi = \phi_J) \propto \sigma.\dot{\gamma} \sim \dot{\gamma}^{y_\gamma}.\dot{\gamma} \sim \dot{\gamma}^{1+y_\gamma}$). Since we adapt a more accurate critical point than that used by Hatano, our q exponent is slightly larger than the value reported by Hatano, but still in the same range within the numerical accuracy. Rescaled data for power flux with $\dot{\gamma}^q$, results in a plot in which all curves cross at the single critical jamming point—Main Fig. 7. Now, in order to estimate the other critical exponent, we draw a horizontal line (the horizontal solid line in the Main Fig. 7) which intersects all the curves and the vertical dashed line passing through the jamming point ϕ_J. We find that the distance between the intersection ϕ' for each curve and ϕ_J, shows a power-law behavior $|\phi' - \phi_J| \sim \dot{\gamma}^{q/y}$ with $q/y = 0.75(15)$—see Inset Fig. 7.

Using these two exponents, we find that all data collapse onto a single universal function when suitably rescaled—Fig. 8.

Although the exponents q and y are specific to the corresponding observable which is measured, their ratio $q/y = 1/z\nu$ should be independent of the measured observable. In this sense our obtained exponent $q/y = 0.75(15)$ is larger than that obtained by Hatano as $q/y = 0.42(4)$. The reason is that here we demand a data collapse for a rather wide range of packing fractions far from the critical jamming point. In order to show that our exponents are in perfect agreement with those reported by Hatano, we focused on the critical region and generated more data around the critical point—Fig. 9, left panel. We then estimated the exponent q/y around ϕ_J and find that $q/y = 0.49(10)$ in perfect agreement with that reported by Hatano withing the error bars—Fig. 9, right panel. We have collapsed the average pressure with the same critical exponents.

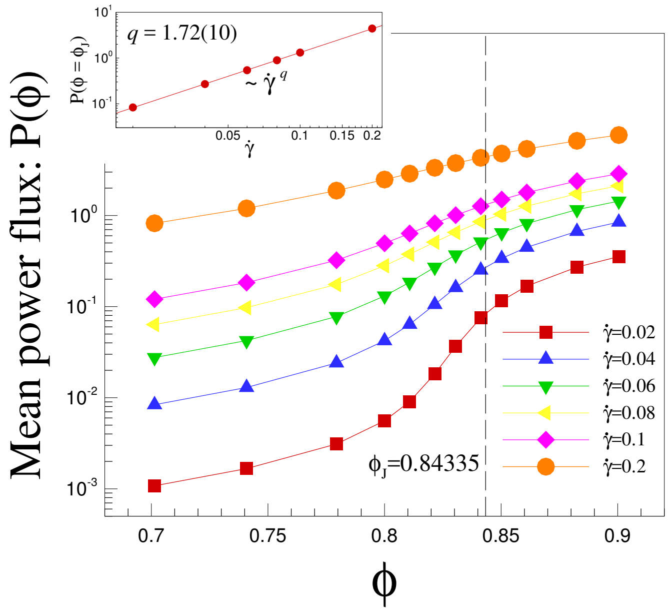

Figure 6: **Mean injection power.** Main: Mean power flux $P(\phi)$ in a system of linear size $L = 30$ as a function of packing fraction ϕ for different shear rates $\dot{\gamma} = 0.02, 0.04, 0.06, 0.08, 0.1$ and 0.2. The vertical dashed line shows the critical packing fraction ϕ_J at which the jamming transition occurs. Inset: Scaling behavior of the mean power flux at the jamming transition point as a function of shear rate, gives the critical exponent $q = 1.72(10)$.

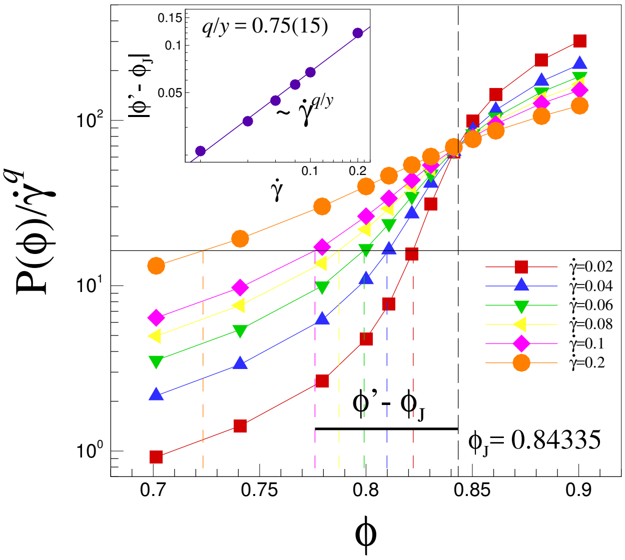

Figure 7: **Semi-scaled mean injection power.** Main: Rescaling of the data along the y-axis in terms of the scaling exponent q obtained in Fig. 6 at ϕ_J. Inset: Scaling behavior of the distance between the intersection ϕ' of the horizontal solid line with each curve and the vertical dashed line passing through ϕ_J.

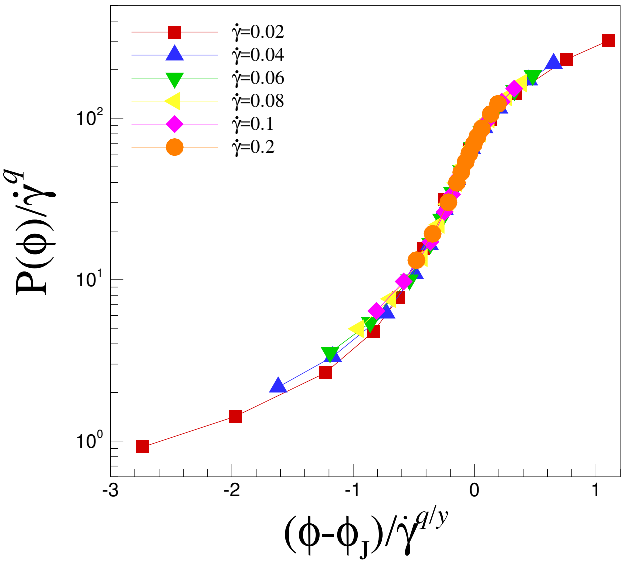

Figure 8: **Collapse of mean power for $|\delta\phi| \leq 10^{-1}$.** Data collapse for the mean power flux by using the exponents obtained in Figs. 6 and 7.

.

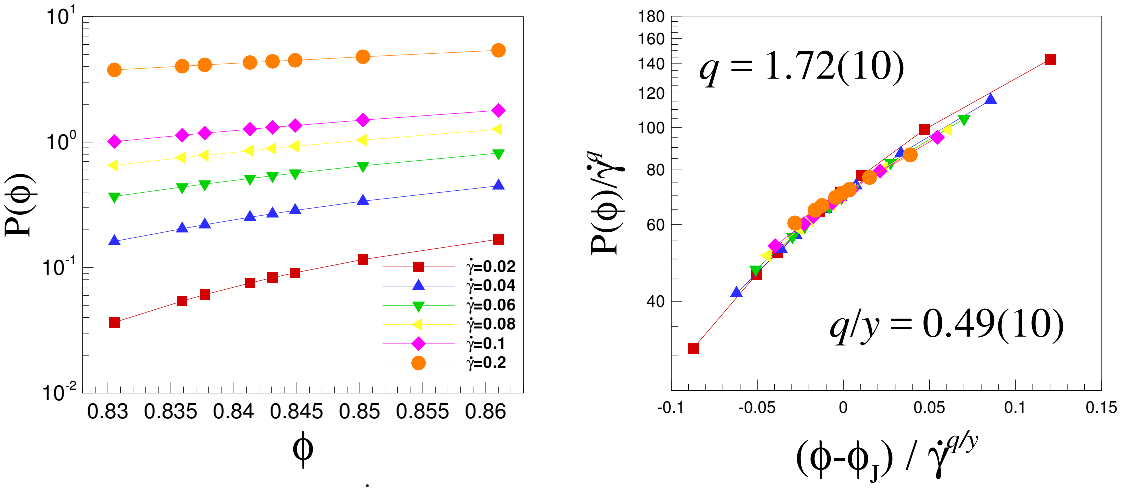

Figure 9: **Collapse of mean power for $|\delta\phi| \leq \mathbf{10^{-2}}$.** Data collapse (right panel) for the mean power flux (left panel) very close to the jamming transition point with modified exponents in close agreement with those reported in [T. Hatano, Progr. Theor. Exp. Phys., 184 143 (2010)].

0.3.2 Comment 2

Referee's comment:

"The authors mention other measurements of effective temperatures in systems of $2D$ and $3D$ frictionless disks that used linear response relations rather fluctuation relations. The authors state on the bottom of page 3 that effective temperatures from fluctuation relations are valid for packing fractions both far and near the jamming packing fraction, whereas those based on linear response are only valid near the jamming point. This statement has not been sufficiently justified by the current studies. In particular, I don't see why the authors state that linear response relations are more valid near jamming onset."

Response: We would like to thank the referee for bringing our attention to this point. As far as our results concern, we show that our proposed FR is valid below and above jamming. However, as one reads from the title of Ref. [14]: "*Effective Temperatures of a Driven System Near Jamming*" it conveys the message that these effective temperatures are valid only near jamming. They also mention in the conclusion of the paper that concept of effective temperature should be valid for the cases where the injected power is fully dissipated by the microscopic fluctuations of particles. As a results of stick-slip regime near jamming they conclude that the concept of the effective temperature must be valid only near jamming where the stick-slip motion is pronounced. Here in our work, we only need rare events to calculate the effective temperature. And as we show, the rare events exist far-below and -above jamming. So our proposed effective temperature does not suffer from such a restriction.

0.3.3 Comment 3

Referee's comment:

"Related to point 2, it would be great if the authors could measure one of the previously studied effective temperatures (defined from linear response) and compare the scaling collapse to that of T_e. This measurement would address the extent to which the assumption of linear response affects the scaling behavior of power or stress fluctuations near ϕ_J."

Response: This is indeed a crucial point to compare all the proposed effective temperatures in the literature. There are only 5 effective temperatures proposed by Ono *et al.* [14] and another one by Haxten and Liu [15] and several others elsewhere [16, 17]. However, a conclusive comparison will take a longer time than the strict time window given by Nature. Could you please possibly allow us to consider this urgent matter be addressed in the near future?

Bibliography

[1] T. Hatano, *J. Phys. Soc. Jpn.* **76** 023001 (2007).

[2] T. Hatano, *J. Phys. Soc. Jpn.* **77** 123002 (2008).

[3] T. Hatano, *Progr. Theor. Exp. Phys.* **184** 143 (2010).

[4] C. E. Maloney, *Europhys. Lett.* **111** 28001 (2015).

[5] G. Gallavotti and E.G. D. Cohen, *Phys. Rev. Lett.* **74**, 2694 (1994).

[6] G. Gallavotti and E.G. D. Cohen, *J. Stat. Phys.* **80**, 931 (1995).

[7] G. E. Crooks, *Phys. Rev. E* **60**, 2721 (1999).

[8] C. Jarzynski, *Phys. Rev. Lett.* **78**, (1997).

[9] U. M. B. Marconi, A. Puglisi, L. Rondoni, and A. Vulpiani, *Phys. Rep.* **461** 111 (2008).

[10] D. J. Evans, E. G. D. Cohen, and G. P. Morriss, *Phys. Rev. Lett.* **71** 2401 (1993).

[11] V. Y. Chernyak, M. Chertkov, and C. Jarzynski *J. Stat. Mech.* **2006** 08001 (2006).

[12] R. Garcia-Garcia, V. Lecomte, A. B. Kolton, and D. Dominguez *J. Stat. Mech.* **2012** 02009 (2012).

[13] D. J. Evans and D. J. Searles, *Phys. Rev. E* **50** 1645 (1994).

[14] I. K. Ono *et al.*, *Phys. Rev. Lett.* **89**, 095703 (2002).

[15] T. K. Haxton and A. J. Liu, *Phys. Rev. Lett.* **99**, 195701 (2007).

[16] C. Song and P. Wang and H. A. Makse, *PNAS* **102** 2299 (2004).

[17] C. S. O'Hern, A. J. Liu, and S. R. Nagel *Phys. Rev. Lett.* **93** 165702 (2004).

REVIEWERS' COMMENTS:

Reviewer #1 (Remarks to the Author):

The authors have adequately addressed all of the comments included in my original review. In particular, they described how their measurements of critical exponents for the power fluctuations relate to those measured previously by Vagberg and Hatano for the pressure and stress.
I now recommend publication of the manuscript.

Reviewer #2 (Remarks to the Author):

The authors have satisfactorily addressed my concerns. I also note that several of the technical objections of reviewer 3 were similar to my own technical objections (role of inertia, framing the discussion in terms of previously known fluctuation relations), and they have addressed these. I strongly recommend publication.

V. REVIEWERS' COMMENTS (SECOND ROUND)

A. Reviewer 1 (Remarks to the Author)

The authors have adequately addressed all of the comments included in my original review. In particular, they described how their measurements of critical exponents for the power fluctuations relate to those measured previously by Vagberg and Hatano for the pressure and stress. I now recommend publication of the manuscript.

B. Reviewer 2 (Remarks to the Author)

The authors have satisfactorily addressed my concerns. I also note that several of the technical objections of reviewer 3 were similar to my own technical objections (role of inertia, framing the discussion in terms of previously known fluctuation relations), and they have addressed these. I strongly recommend publication.

VI. RESPONSE TO REFEREE 1

We would like to thank the reviewer for his/her careful reading of our manuscript. His/her suggestions have improved our manuscript and we appreciate his/her recommendation that our manuscript be accepted for publication in Nature Communications.

VII. RESPONSE TO REFEREE 2

We would like to thank again for the positive and constructive attitude of the referee. We are glad that our response has satisfactorily convinced the reviewer's concerns and we appreciate his/her recommendation that our manuscript be accepted for publication in Nature Communications.

---

[1] T. Hatano, *J. Phys. Soc. Jpn.* **76**, 023001 (2007).

[2] T. Hatano, *J. Phys. Soc. Jpn.* **77**, 123002 (2008).

[3] T. Hatano, *Progr. Theor. Exp. Phys.* **184**, 143 (2010).

[4] C. E. Maloney, *Europhys. Lett.* **111**, 28001 (2015).

[5] G. Gallavotti and E.G. D. Cohen, *Phys. Rev. Lett.* **74**, 2694 (1994).

[6] G. Gallavotti and E.G. D. Cohen, *J. Stat. Phys.* **80**, 931 (1995).

[7] G. E. Crooks, *Phys. Rev. E* **60**, 2721 (1999).

[8] C. Jarzynski, *Phys. Rev. Lett.* **78**, (1997).

[9] U. M. B. Marconi, A. Puglisi, L. Rondoni, and A. Vulpiani, *Phys. Rep.* **461**, 111 (2008).

[10] D. J. Evans, E. G. D. Cohen, and G. P. Morriss, *Phys. Rev. Lett.* **71**, 2401 (1993).

[11] V. Y. Chernyak, M. Chertkov, and C. Jarzynski *J. Stat. Mech.* **2006**, 08001 (2006).

[12] R. Garcia-Garcia, V. Lecomte, A. B. Kolton, and D. Dominguez *J. Stat. Mech.* **2012**, 02009 (2012).

[13] D. J. Evans and D. J. Searles, *Phys. Rev. E* **50**, 1645 (1994).

[14] I. K. Ono *et al.*, *Phys. Rev. Lett.* **89**, 095703 (2002).

[15] T. K. Haxton and A. J. Liu, *Phys. Rev. Lett.* **99**, 195701 (2007).

[16] C. Song and P. Wang and H. A. Makse, *PNAS* **102**, 2299 (2004).

[17] C. S. O'Hern, A. J. Liu, and S. R. Nagel *Phys. Rev. Lett.* **93**, 165702 (2004).
